# Best practices along the kidney transplantation clinical journey

**DOI:** 10.3389/frtra.2026.1779662

**Published:** 2026-04-13

**Authors:** Sara Machado, Stergios Bobotis, Alexander Loupy, Gillian Divard, Valentin Goutaudier, Andreas Pascher, Philipp Houben, Jacopo Romagnoli, Filippo Paoletti, Daniel Casanova, Sofia Zarraga, Mehmet Ergisi, Bryan Ooi, Zoe-Athena Papalois, Elias Mossialos, Vassilios Papalois

**Affiliations:** 1 Brown University, Providence, RI, United States; 2 Maidstone and Tunbridge Wells NHS Trust, Maidstone, United Kingdom; 3 Universite Paris Cite, Paris, France; 4 Universitat Munster, Münster, Germany; 5 Fondazione Policlinico Universitario Agostino Gemelli IRCCS, Rome, Italy; 6 Hospital Universitario Marques de Valdecilla, Santander, Spain; 7 Hospital Universitario Cruces, Barakaldo, Spain; 8 Imperial College London, London, United Kingdom; 9 The London School of Economics and Political Science, London, United Kingdom

**Keywords:** best practices, CKD—chronic kidney disease, kidney donation, kidney recipient, kidney transplantation

## Abstract

**Background:**

Kidney transplantation (KTx) practices vary across healthcare systems, yet the operational components of best practice (BP) along the clinical pathway remain incompletely defined. This study aimed to identify key best practice elements across the kidney transplantation journey in four European countries.

**Methods:**

A mixed-methods study was conducted across France, Germany, Italy, and Spain. A structured survey (*n* = 253 respondents, including patients, living donors, nephrologists, transplant surgeons, transplant coordinators, and hospital administrators) assessed clinical practice and patient experience across four domains: CKD management, kidney donation and transplantation, transplant recipient care, and service governance. Semi-structured focus group interviews were performed in each country to contextualise survey findings. Ethics approval was obtained in accordance with national requirements.

**Results:**

Key elements for best practices along the KTx clinical journey were identified: (1) development of protocols to standardise the variable monitoring of CKD, to minimize urban-rural differences in clinical practice due to limited resources and follow-up care; (2) enhanced primary care training and targeted resource allocation to diagnose and monitor early-stage CKD; (3) donor coordination and promotion of living donation, addressing gaps in patient awareness and access to care; (4) development of communication protocols on living donation; (5) implementation of targeted patient and donor educational campaigns on living donation; (6) enhanced post-transplant follow-up care by nephrologists; (7) integration of quality-of-life assessments and psychological donor support post-transplantation; (8) increased availability of transplant coordinators to promote equitable resource allocation and the adoption of innovative practices; (9) streamlined governance structures along clinical journey; and (10) equitable funding models with consistent reimbursement policies across patient groups.

**Conclusions:**

This study provides a cross-national, mixed-methods framework for strengthening equity, coordination, and quality in kidney transplantation. Addressing variability in monitoring pathways, referral structures, patient-centred outcomes, and workforce capacity may enhance implementation of international transplantation guidelines and improve patient and donor outcomes.

## Introduction

Kidney transplantation is the preferred treatment for end-stage kidney disease (ESKD), offering superior survival outcomes, improved quality of life, and reduced long-term healthcare costs compared to dialysis (1–3). International guidelines from organisations such as the Kidney Disease: Improving Global Outcomes (KDIGO), the European Renal Best Practice (ERBP), the British Transplantation Society (BTS), the American Society of Transplantation (AST), and the European Society for Organ Transplantation (ESOT) have been instrumental in formulating comprehensive clinical practice guidelines that aim to standardize and optimize patient care in kidney transplantation (4–8). Challenges persist throughout the kidney transplantation pathway, from early-stage chronic kidney disease (CKD) monitoring to post-transplant care. Variability in clinical practices, donor availability, healthcare governance, and financial sustainability continues to impact patient access, outcomes, and equitable resource allocation (9, 10).

This study assesses the implementation of existing guidelines along the kidney transplantation clinical journey, from chronic kidney disease (CKD) prevention to long-term post-transplant care, and outlines a set of best practices recommendations.

## Methods

This study employed a mixed-methods approach to infer best practices along the kidney transplantation clinical journey across four European countries—France, Germany, Italy, and Spain. The study, built upon existing guidelines, was structured into two primary components: a quantitative survey capturing clinical practices and patient experiences and qualitative focus group interviews providing deeper insights into key challenges and best practice implementation.

### Study population & setting

Data were collected from individual stakeholders across transplant centres in each of the four participating countries. Participants included patients, living kidney donors, transplant surgeons, nephrologists, transplant coordinators, and hospital administrators, representing a comprehensive cross-section of the kidney transplantation clinical pathway.

### Quantitative data collection & survey design

A structured survey tool was designed to identify best practices in the kidney transplantation clinical journey (Supplementary Material). The survey was developed through a literature review of international guidelines (including KDIGO, European Commission CD-P-TO, and British Transplantation Society guidelines) and expert validation, including review by an international panel of transplant specialists, nephrologists, transplant coordinators, and patient representatives. The survey was categorised into 4 thematic sections: CKD, Kidney Donation and Transplantation, Transplant Recipients, and Management of Donation and Transplantation Service. Pilot testing was performed, where a subset of participants completed the survey twice to assess consistency and clarity. The final survey was distributed electronically via the Qualtrics platform, with translations provided in German and Spanish, where required by ethical regulations. Local champions at each transplant centre facilitated survey distribution via targeted email and clinic outreach. Participants responded to questions appropriate to their role and experience—for example, patients and donors responded to items on education and support, while clinicians and coordinators addressed questions relating to clinical protocols, governance, and service delivery.

### Qualitative data collection & focus groups

To complement survey findings, semi-structured focus group interviews were conducted in each participating country. Participants included patients, living donors, nephrologists, transplant surgeons, transplant coordinators, and hospital administrators, purposively selected to ensure representation across stakeholder groups and transplant centres. Recruitment was facilitated by designated local leads (“local champions”) at each centre, who identified and invited eligible participants. Participation was voluntary and uncompensated, and all participants provided informed consent prior to participation.

Focus groups were conducted online and moderated by a member of the research team with clinical and transplantation expertise. A semi-structured interview guide, developed on the basis of preliminary survey findings and established guideline domains, was applied consistently across countries to ensure thematic comparability. Discussions lasted approximately 60–90 min and were conducted in the local language. Audio recordings were transcribed verbatim and, where necessary, translated into English to enable cross-country thematic analysis. Separate focus group discussions were conducted in each participating country (Germany, Italy, Spain, and France), with participants recruited locally through transplant centres and professional networks. Participant composition varied across countries depending on local stakeholder availability and recruitment pathways. Only stakeholder role and transplant centre affiliation were recorded for focus group participants; no additional demographic variables were systematically collected.

### Data analysis

#### Quantitative analysis

Survey data were analysed using descriptive statistics, summarising responses across key thematic areas. Categorical variables were reported as percentages and frequencies, while Likert scale responses were analysed using mean scores.

#### Qualitative analysis

Focus group transcripts underwent thematic analysis by three independent reviewers (SB, SM, VP). An initial analytic framework was developed deductively based on the four study domains and the corresponding survey sections, ensuring alignment between qualitative themes and the quantitative constructs assessed. The framework was iteratively refined through transcript review, with themes agreed through reviewer discussion. Qualitative themes were then triangulated with relevant survey questions and findings to contextualise observed practice variation and inform best practice recommendations.

### Ethics approval

Ethics approval was obtained through a two-tiered process. The study received conditional ethics approval from the London School of Economics (LSE) Ethics Review Board, after which national leads coordinated approvals from relevant local ethics committees in each country if needed (Supplementary Material). In Germany, local institutional processes confirmed that additional ethics approval was not required and that LSE approval was sufficient. Ethics approvals covered both the survey and focus group components, ensuring compliance with country-specific human subjects research guidelines.

## Results

### Study population

A total of 253 participants completed the survey, comprising 93 nephrologists, 35 transplant surgeons, 48 patients, 20 transplant coordinators, 19 hospital administrators, and 12 living kidney donors. The questionnaire consisted of 87 items, including both multiple-choice and open-text questions, designed to capture perspectives on chronic kidney disease (CKD) monitoring, donor coordination, living donation, and post-transplant care. The median survey completion time was 15 min (IQR 10–21).

In addition, a total of 47 participants took part in the focus group discussions across the four participating countries. Table 1 presents the characteristics of the 47 focus group participants across the four participating countries (Germany, Italy, Spain, and France), stratified by stakeholder role. Italy and Spain contributed the largest number of participants (*n* = 17 and *n* = 16, respectively), followed by France (*n* = 11) and Germany (*n* = 3). Transplant surgeons formed the largest stakeholder group overall (*n* = 21), with particularly strong representation from Spain (*n* = 14). Nephrologists were the second most represented group (*n* = 8), predominantly from France (*n* = 4) and Italy (*n* = 3). Transplant coordinators (*n* = 7) and hospital administrators (*n* = 5) were also represented across multiple countries, while recipients/patients (*n* = 4) and living donors (*n* = 2) participated in smaller numbers, with living donor representation limited to Italy only.

**Table 1:** Characteristics of focus group participants by country and stakeholder role.

Country	Germany	Italy	Spain	France	Total
Total participants (*n*)	3	17	16	11	**47**
By stakeholder role					
Transplant surgeon	1	4	14	2	**21**
Transplant coordinator	0	3	2	2	**7**
Nephrologist	1	3	0	4	**8**
Living donor	0	2	0	0	**2**
Recipient (patient)	1	2	0	1	**4**
Hospital administrator	0	3	0	2	**5**

Number of participants (*n*)

Further detail on the focus group sessions conducted in each country is provided in Supplementary Table 4. Germany contributed a single session held in November 2024 at University Hospital Münster, moderated by Dr. Philipp Houben. Italy hosted two sessions in July 2023 across six transplant centres (Gemelli Rome, Padova, Bologna, Milano Policlinico, Milano Niguarda, and Bari), moderated by Professor Jacopo Romagnoli and Dr. Filippo Paoletti. France similarly conducted two sessions in November 2024 across four centres (CHU de Nantes, CHU de Bordeaux, Hôpital Saint-Louis APHP Paris, and Hôpital Necker APHP Paris), moderated by Dr. Gillian Davard. Spain held two sessions in September and October 2023, primarily at Hospital Universitario Cruces, with Dr. Sofía Zárraga serving as moderator. Across all countries, sessions were conducted at nationally representative transplant centres, ensuring diversity of institutional perspectives within each participating country.

### Main themes and best practice recommendations

The following set of best practice recommendations summarise the main elements identified across the quantitative and qualitative components of the study, across the four thematic areas. The key elements leading to these recommendations are discussed below, referencing specific Questionnaire results (Supplementary Material) as appropriate.

The main themes are (1) Addressing Variability in CKD Monitoring and Early-Stage Management, (2) Optimising Donor Coordination, (3) Living Donation Promotion, (4) Patient Awareness, Education, and Access to Resources, (5) Improving Post-Transplant Patient Care, (6) Psychological donor support & Quality-of-life assessments, (7) Strengthening Healthcare Infrastructure and Governance, (8) Streamlining Governance Structures, and (9) Equitable Funding & Reimbursement Models, which shaped the following best practice recommendations.

#### Best practice recommendations

1. *Develop standardized CKD monitoring protocols to minimise regional disparities in clinical practice.*
2. *Enhance primary care training and resource allocation to improve early CKD diagnosis and management.*
3. *Improve donor coordination and living donation promotion by addressing gaps in patient awareness.*
4. *Implement structured communication protocols to improve living donation discussions.*
5. *Develop targeted educational campaigns for both patients and donors.*
6. *Enhance post-transplant follow-up care through nephrologist-led monitoring.*
7. *Integrate quality-of-life assessments and psychological support into post-transplant care.*
8. *Expand the availability of transplant coordinators to improve resource allocation and innovation adoption.*
9. *Streamline governance structures to enhance the efficiency of the clinical transplant journey.*
10. *Develop equitable funding models with consistent reimbursement policies across patient groups.*

### CKD monitoring and donor referral systems

#### Addressing variability in CKD monitoring and early-stage management

Chronic kidney disease (CKD) represents a significant burden in transplant medicine, yet its monitoring and management remain inconsistent across healthcare systems. Survey data indicated substantial variability in early detection and monitoring strategies, referral pathways, and follow-up protocols (Q1, Q7–8, Supplementary Material). Focus group discussions reinforced these findings, with participants from rural regions emphasising delays in nephrology referrals, resource limitations, and inconsistent application of best-practice guidelines. These results align with existing literature, where disparities in nephrology access have been linked to delayed interventions and increased dialysis dependence at the time of transplantation (4, 11, 12).

International frameworks, including the KDIGO and European Renal Best Practice guidelines, advocate for standardised CKD care pathways to address these disparities (2, 4). However, our findings suggest that implementation remains fragmented, with primary care providers, who are involved in the regular care of patients newly diagnosed with CKD, often lacking the necessary training to detect and manage early-stage disease (Q5). Focus group discussions highlighted that early intervention strategies were frequently absent, largely due to competing clinical priorities and limited specialist support (Q20, German focus group). This is supported by research indicating that education programs targeting primary care physicians can significantly improve early CKD diagnosis rates (13).

Survey data further revealed that while general practitioners (GPs) were frequently involved in CKD management, access to educational resources for both patients and clinicians was limited, with many patients reporting difficulty accessing CKD-related information (Q4). Similarly, monitoring protocols varied widely, with only 19% of respondents rating CKD monitoring frequency as adequate, while 33% reported monitoring as infrequent or far too infrequent (Q6, Q7), highlighting the need for greater standardisation in follow-up practices. Studies suggest that early nephrology referrals and structured CKD monitoring programs improve patient outcomes and reduce late-stage CKD complications (11, 13).

Although standardised national guidelines provide a framework for CKD monitoring, local adaptations remain necessary to address regional healthcare disparities and resource availability (9, 13).

### Optimizing donor coordination

Effective donor coordination is essential for maximising kidney transplantation rates. Survey data indicated that referral systems for deceased donors were generally well-established, with most respondents (>70%) rating them as very or highly effective across different types of donation, including donation after brain death (DBD) and donation after circulatory death (DCD) (Q22, Q23). A key strength of these systems was their strong reliance on well-defined clinical criteria, particularly for DBD and DCD referrals, where the majority of respondents reported high or exceptional reliance on established medical guidelines (Q24, Q25). These findings align with literature emphasising that structured and efficient referral pathways are crucial for timely donor identification and organ procurement (14–16).

Beyond referral pathways, survey respondents described coordination and communication among all transplant specialties as very effective, including collaboration between organ-specific retrieval teams (Q52) and between donor coordinators and other healthcare professionals (Q35). However, despite the perceived efficiency in coordination, focus group findings highlighted significant inefficiencies in donor referral pathways and organ allocation systems. Many healthcare professionals emphasised that donor coordination frameworks vary between transplant centres, with some institutions implementing robust pre-donation counselling and structured follow-up, while others lack well-defined donor management strategies. The inconsistency in donor evaluation protocols has been previously reported as a barrier to increasing donor pool utilization (15).

While existing referral systems are largely effective, moderate effectiveness ratings reported by a significant proportion of centres suggest room for improvement, particularly in optimising donor identification, enhancing pre-donation counselling, and ensuring equitable access to transplantation opportunities. Studies suggest that early and transparent education about donation options significantly impacts donor willingness and participation rates (10, 13). Ensuring that donor referral systems are supported by comprehensive and patient-centred communication strategies may further enhance their impact and improve organ donation rates across different transplant settings.

### Living donation pathways and patient access to transplantation

#### Living donation promotion

Living donation remains an underutilised strategy despite its well-documented benefits. Focus group discussions underscored the late introduction of living donor options in the CKD trajectory, which reduces the likelihood of pre-emptive transplantation. This aligns with existing research showing that earlier integration of living donor education leads to higher donor engagement and transplant success rates (17–19).

A major barrier identified in the qualitative dataset was the absence of structured communication protocols between transplant professionals and potential donors. Transplant coordinators expressed concerns that donor apprehension often arises from misconceptions about eligibility, surgical risks, and financial implications, which are inadequately addressed by current patient education materials. Studies have shown that tailored communication strategies, including personalised counselling and culturally adapted informational resources, can significantly improve donor willingness and participation (6, 18, 20, 21).

Best practice guidelines suggest that living donation discussions be incorporated into nephrology and transplant consultations at the earliest possible stage, ensuring patients and their families are aware of all available options (5–7). By enhancing structured counselling, reducing misinformation, and promoting transparent discussions about the benefits and risks of living donation, transplantation programs can increase donor engagement and expand the living donor pool (17, 20, 21).

### Patient awareness, education, and access to resources

Patient awareness and access to transplantation-related resources remain significant barriers to equitable care. Focus groups revealed disparities in how patients receive information about kidney transplantation, with many reporting limited access to educational materials and inconsistent guidance from healthcare providers. Survey respondents indicated that consultations most often addressed medication adherence and potential surgical complications, whereas emotional preparation for surgery and the creation of patient support structures were the least discussed (Q13). This finding aligns with literature showing that comprehensive transplant education should address both clinical and psychosocial factors to improve patient engagement and long-term adherence (13, 14, 22, 23).

While access to resources such as transplant coordinators (Q50), patient groups (Q4), and pre-transplant educational materials (Q11) was generally rated as moderately to very effective, participants emphasised the need for centralised, multilingual, and patient-friendly educational tools to bridge gaps in knowledge and improve decision-making. The complexity of the donation and evaluation process was a recurring concern, with German focus group participants suggesting clearer, standardised checklists for both donors and recipients. These observations are consistent with studies highlighting that educational disparities contribute to lower transplant rates in underserved populations, reinforcing the need for culturally adapted, patient-centred interventions (21–24).

Emerging evidence supports the role of interactive digital resources and peer-supported education in improving patient engagement. Digital education platforms have been shown to enhance transplant knowledge and preparedness, particularly among younger patients (14, 21), while peer support networks can increase confidence and willingness to pursue transplantation, especially among hesitant candidates (14, 20). The KDIGO (2020) guidelines recommend integrating multilingual digital resources and standardised transplant education materials into routine pre-transplant counselling, ensuring that all CKD patients, regardless of socioeconomic background or geographic location, have access to high-quality educational tools (1).

To address these challenges, transplantation programs should develop standardised, accessible educational platforms, expand online resources, produce multilingual patient guides, and strengthen community-based education initiatives. Such measures will be critical in reducing disparities in transplantation access, improving decision-making, and ultimately enhancing post-transplant outcomes (13, 14, 21–24).

### Quality of life and comprehensive patient support

#### Improving post-transplant patient care

Despite significant advancements in transplantation medicine, post-transplant care remains highly variable, with notable differences in nephrologist-led follow-up, psychological support, and medication adherence. In both the short- and long-term post-transplant period, patient management is predominantly overseen by nephrologists and transplant surgeons (Q65–66). However, the frequency of follow-up visits declines over time, with most patients receiving weekly or bi-monthly follow-ups in the first three months, and even less frequent consultations thereafter (Q72). While KDIGO Guidelines (2020) recommend lifelong nephrologist follow-up to mitigate risks such as chronic allograft nephropathy, rejection, and cardiovascular complications (1), the observed reduction in structured follow-up raises considerations about whether long-term monitoring remains optimal for all patients (3, 25). There is also an ongoing discussion about the role of early primary care physician integration, which could complement specialist-led care by improving holistic, long-term management strategies.

Qualitative data from focus group discussions further highlighted concerns about the adequacy of long-term support, particularly in relation to medication adherence, mental health, and post-transplant lifestyle adjustments. Given that non-adherence to immunosuppressive therapy is recognised as a significant contributor to graft failure (26–29), strengthening follow-up protocols and patient engagement strategies may be beneficial in mitigating this risk. The literature suggests that structured education programs and adherence monitoring tools can help sustain long-term medication compliance and improve transplant outcomes (26–30).

### Psychological donor support & quality-of-life assessments

Beyond recipient care, focus group data indicated areas for improvement in post-donation support, with many living donors reporting insufficient engagement in follow-up care and expressing concerns about their long-term health status. Several donors described experiencing anxiety regarding their kidney function, feeling that post-donation support ends too soon after surgery. These concerns align with broader research findings indicating that living donors may face long-term psychological distress and uncertainty about their health (3, 15, 18). While some post-donation support structures exist, their availability and consistency vary across transplant programs, highlighting the need for standardised post-donation follow-up models that comprehensively address both clinical and psychosocial needs (4, 31).

The integration of quality-of-life (QoL) assessments into post-transplant care remains inconsistent. Survey data revealed that only 13% of transplant centres consistently collect patient-reported QoL data (Q74), while 44% collect it sporadically and 43% do not collect it at all. Similarly, the use of patient-reported outcome measures (PROMs) and donor-reported outcome measures (DROMs) is limited, with PROMs collected consistently in only 13% of centres, sometimes in 39%, and never in 49% (Q75). DROMs are even less integrated, with 55% of centres not collecting any donor-reported data, while 30% collect them sporadically (Q76). This reflects findings from international studies that show PROMs and QoL tools are more commonly used in clinical trials than in real-world practice, largely due to resource constraints and the absence of standardized implementation frameworks (15, 32).

The lack of systematic QoL monitoring limits the ability to evaluate long-term patient and donor well-being, underscoring the need for broader implementation of PROMs and DROMs to inform post-transplant care strategies effectively (1, 33, 34) Studies indicate that low QoL scores post-transplantation correlate with increased hospitalisation and graft failure, reinforcing the need for early and structured QoL assessments (33, 34) Additionally, the American Society of Transplantation (AST) and European Renal Best Practice (ERBP) guidelines advocate for the routine collection of QoL data as part of comprehensive transplant care, yet challenges remain in ensuring widespread adoption due to clinical workload and reimbursement barriers (5, 7). Addressing these gaps through integrated digital tools, standardized questionnaires, and policy-driven incentives may facilitate the adoption of QoL assessments and enhance post-transplant patient care (33, 34).

### Transplant governance, infrastructure, and financial sustainability

#### Strengthening healthcare infrastructure and governance

Transplant coordinators play a vital role in facilitating donor identification, recipient matching, and post-operative care, yet their availability varies significantly across transplant centres (Q50). Survey data revealed that while transplant coordinators were generally perceived as accessible, a notable proportion of centres reported moderate or limited availability, indicating potential workforce constraints (Q51). Workforce shortages, particularly in France, were identified as a key bottleneck in the transplant process, affecting donor referral efficiency and organ allocation logistics.

Focus groups emphasized that transplant coordinators are integral to optimising transplantation pathways, yet several centres reported that coordinators are overburdened, leading to delays in organ allocation. This sentiment is reinforced by international literature, which has linked higher transplant coordinator staffing levels with improved patient outcomes, shorter waitlist times, and reduced organ discard rates (15, 35).

Despite recognition of their importance, transplant coordinator workforce shortages remain a challenge globally. Studies have highlighted persistent recruitment and retention issues, with burnout and limited professional development opportunities contributing to high turnover rates (35). The KDIGO Best Practices guidelines advocate for increasing the number of trained transplant coordinators, citing evidence that a low coordinator-to-patient ratio correlates with suboptimal transplant outcomes. Expanding transplant coordinator roles and ensuring sustainable workforce planning will be crucial to enhancing efficiency and equity in kidney transplantation services.

### Streamlining governance structures

The governance of kidney transplantation varies considerably across transplant centres, with differences in regulatory oversight, data integration, and coordination between medical teams contributing to variations in practice. Survey data assessing the effectiveness of health authorities in overseeing transplantation governance revealed a broad range of perceptions, with some respondents rating governance structures as effective, while others considered them only moderately or slightly effective (Q87). These findings suggest differences in governance implementation across regions, which was further reinforced by focus group discussions highlighting variability in donor allocation policies and communication challenges among transplant stakeholders. Literature supports that a harmonized governance framework can enhance transplant efficiency and promote equity in organ allocation (1, 3, 5–8).

One notable area of variation was protocols for determining death in potential donors, particularly in donation after brain death (DBD). While national guidelines were commonly used, regional and local protocols were also present, resulting in differences in practice across transplant centres (Q29). These protocols were developed with input from multiple stakeholders, including donation/transplantation authorities, donor coordinators, local clinicians, and organ procurement teams, reflecting a multidisciplinary approach that fosters comprehensive and adaptable governance structures (Q30). Beyond death determination, protocol efficiency for obtaining family consent for donation after circulatory death (DCD), monitoring and optimizing potential donors after brain death (DBD), and determining the timing of organ procurement varied across centres. While many centres reported these protocols as effective, some identified challenges in ensuring timely donor management and organ retrieval, highlighting areas for potential improvement (Q36, Q38, Q39). These findings align with broader research emphasizing the importance of standardized, evidence-based donor management protocols in maximizing organ utilization and post-transplant success (36, 37).

While national guidelines provide a foundation for consistency, local adaptations remain necessary to account for regional healthcare structures and resource availability. A balanced approach that integrates standardization with contextual flexibility may be key to ensuring both equity and efficiency in organ donation and transplantation governance (1, 9).

Additionally, survey data indicated variation in the adoption of electronic medical records (EMRs) across transplant centres (Q86), with some facilities fully integrating digital tracking for transplant recipients, while others continue to rely on paper records. Literature supports the role of comprehensive EMR systems in improving patient outcomes, as they facilitate real-time donor-recipient matching, predictive risk stratification, and streamlined post-transplant follow-up (37–39). However, barriers to widespread implementation persist, including technical limitations, cost concerns, and regulatory hurdles, which may impact the feasibility of universal adoption (40).

The debate surrounding centralization of transplant governance remains complex. While a standardized national framework can promote best practices and consistency, some concerns persist regarding the potential loss of flexibility in adapting to regional healthcare needs. Studies suggest that a degree of local decision-making may be beneficial, allowing transplant programs to tailor services to specific patient populations (9, 41). Nonetheless, there is broad consensus that greater alignment of national policies, improved inter-centre communication, and enhanced regulatory efficiency would contribute to more equitable and effective transplantation governance (4–8, 41).

### Equitable funding & reimbursement models

Funding and reimbursement structures play a crucial role in shaping the accessibility and sustainability of kidney transplantation services. Survey data indicated that financial incentives and penalties for providers are present but vary in scope. Specifically, financial incentives or penalties were most commonly tied to operational targets (e.g., wait times) and patient-reported outcomes (e.g., patient satisfaction), whereas fewer centers applied them to clinical outcomes like mortality rates or cost-efficiency targets (Q85). These findings suggest that while financial mechanisms are used in some settings, their influence on provider behaviour and resource allocation remains inconsistent. Literature suggests that incentive-driven funding models can influence healthcare delivery, yet their impact on long-term transplant outcomes is still debated (42–44).

The adequacy of Diagnosis-Related Group (DRG) reimbursement received mixed responses, with some centres finding it sufficient to cover the full scope of transplant-related expenses, including post-transplant care and pharmaceuticals, while others indicated it was only somewhat adequate or, in some cases, inadequate (Q84). Financial support mechanisms for both dialysis patients and transplant recipients were also perceived as moderate but with room for improvement (Q83). Nonetheless, financial models did not fundamentally dictate clinical decision-making (Q81). This reflects broader concerns in the literature, which highlights that current DRG-based funding models may not fully capture the complexity and long-term costs associated with transplantation, particularly in centres handling high-risk or resource-intensive cases (41–44). Across all countries, financial constraints were reported, with France and Italy particularly noting challenges in funding innovation and expanding access to advanced therapies. These findings reinforce calls for revised reimbursement frameworks that better align with actual resource utilization and patient needs. A well-structured funding model is essential to ensuring equitable access to transplantation services while also supporting innovation and high-quality patient care (9, 42–44).

While standardized reimbursement models contribute to financial predictability, balancing national funding structures with centre-specific financial needs remains a challenge. A harmonized yet flexible funding model may help optimize resource allocation while maintaining equitable access to transplantation services (4–8, 41–44).

## Alignment with and implications for international guidelines

International guidelines from KDIGO, ERBP, AST, and ESOT provide comprehensive clinical frameworks for CKD management, transplant candidacy, donor evaluation, and post-transplant follow-up (1–8). Our findings largely align with these recommendations but highlight important implementation gaps that persist across healthcare systems. For example, while KDIGO endorses risk-stratified CKD monitoring and early transplant planning, our data demonstrate variability in monitoring frequency and referral thresholds, suggesting that clearer operational benchmarks and audit indicators may enhance guideline uptake in routine practice (1, 2, 4).

Similarly, international guidance increasingly recognises the importance of patient-centred outcomes and long-term donor follow-up (1, 3, 5, 7); however, the limited integration of PROMs and DROMs observed in our study indicates that practical implementation strategies and minimum data standards remain underdeveloped. Incorporating explicit guidance on routine outcome measurement timepoints and registry linkage may strengthen the translational impact of these recommendations.

In addition, although European frameworks emphasise the role of donor coordinators and structured governance (8, 15, 45, 46), the observed heterogeneity in coordination practices suggests that workforce planning benchmarks and process indicators could be more clearly articulated within future guideline iterations. Taken together, our results complement existing clinical guidance by identifying system-level and operational domains—monitoring pathways, referral triggers, patient-reported outcomes, and workforce governance—where enhanced specification may improve consistency, equity, and real-world implementation across transplantation programmes.

### Limitations

This study has limitations related to its geographic scope. Data were collected from transplant centres in four European countries (France, Germany, Italy, and Spain) and may not fully capture variation in clinical practice across other European regions, including Eastern and Northern Europe, where differences in healthcare organisation, resources, and cultural contexts may influence kidney transplantation pathways. Future research should extend this work across a wider range of European healthcare systems to better characterise regional variation and its underlying drivers. In addition, the distribution of respondents across stakeholder groups was uneven. While nephrologists and transplant clinicians were well represented, fewer responses were obtained from living kidney donors and some other stakeholder groups, which may limit the extent to which certain perspectives are fully captured. Nevertheless, the inclusion of multiple professional roles and patient groups provides a broad overview of practice patterns across the clinical pathway. Future studies should prioritise targeted recruitment strategies to enhance representation of under-represented groups, particularly living donors.

Participant recruitment was facilitated through local advocates and centre-based dissemination, which may have introduced selection bias. Individuals and centres more actively engaged in transplantation practice or quality improvement initiatives may have been more likely to participate, potentially limiting the representativeness of the sample. While this approach enabled efficient recruitment across multiple countries, future studies could mitigate this bias through broader recruitment strategies and more systematic sampling approaches. Finally, although the questionnaire underwent expert review and pre-testing to ensure content validity and clarity, formal psychometric reliability measures (such as internal consistency or test–retest reliability) were not assessed, reflecting the exploratory and multi-domain nature of the instrument.

## Conclusions and policy implications

This comprehensive analysis of kidney transplantation clinical practices across four European countries highlights substantial variability in care delivery and identifies clear opportunities for standardisation and improvement. Our findings underscore the need for coordinated action across the entire transplantation pathway, from early CKD detection to long-term post-transplant care.

At the national level, priority actions include the implementation of standardised CKD monitoring protocols, enhanced primary care training to support earlier diagnosis, timely referral to nephrology services when clinically indicated, and structured communication frameworks for living donation. The establishment of national registries capturing both clinical outcomes and patient-reported measures is essential to support quality improvement and accountability, and should be aligned with European initiatives, including ESOT registries, to enable cross-national benchmarking and harmonised data standards.

At the European level, greater policy harmonisation is required to address cross-border disparities in transplantation practice. Pan-European clinical standards shared educational resources, and collaborative research networks should be strengthened, building on existing models such as the ESOT–UEMS training and accreditation framework.

Effective implementation of these recommendations will require a multi-stakeholder approach involving patients, clinicians, policymakers, and payers. Key priorities include expanding patient education, integrating quality-of-life assessments into routine care, and strengthening post-donation support, including standardised documentation to protect living donors from inappropriate post-donation CKD classification and its associated financial and psychological consequences.

Together, these findings provide a pragmatic framework for improving equity, quality, and coordination in kidney transplantation services. Addressing the observed variability in practice through aligned policy initiatives and clinical leadership has the potential to enhance transplant access, improve patient and donor outcomes, and optimise resource utilisation across Europe and beyond.

## Data Availability

The raw data supporting the conclusions of this article will be made available by the authors, without undue reservation.
