# Supplementary material for: Best practices along the kidney transplantation clinical journey
Source: Front Transplant. 2026 Apr 13;5:1779662. doi: 10.3389/frtra.2026.1779662 (PMC13111577; doi:10.3389/frtra.2026.1779662)
Supplement: Supplementary file 1 [file Table1.docx]

**Table S1.** National leads and study governance structure

| **National Leads** | | |
| --- | --- | --- |
| **Country** | **Lead** | **Deputy** |
| Italy | Professor Jacopo Romagnoli | Dr Filippo Paoletti |
| France | Professor Alexandre Loupy | Dr Valentin Goutaudier, Dr Gillian Divard |
| Germany | Professor Andreas Pascher | Dr Philipp Houben |
| Spain | Professor Daniel Casanova | Dr Sofia Zarraga |

| **Italy** | | | | | | | | |
| --- | --- | --- | --- | --- | --- | --- | --- | --- |
| **Centre** | | | **Hospital** | | | **Local Lead** | | |
| Padova | | | Azienda Ospedaliero-Universitaria di Padova | | | Lucrezia Furian | | |
| Torino | | | Azienda Ospedaliero-Universitaria di Torino ospedale Molinette | | | Luigi Biancone | | |
| Milano | | | Fondazione IRCCS Ca' Granda Ospedale Maggiore Policlinico | | | Piergiorgio Messa/Mariano Ferraresso | | |
| Milano | | | Ospedale Niguarda | | | Enrico Minetti/Luciano De Carlis | | |
| Verona | | | Azienda Ospedaliero-Universitaria di Verona | | | Luigino Boschiero/Gianluigi Zaza | | |
| Bologna | | | Policlinico Sant'Orsola-Malpighi | | | Matteo Ravaioli/Gaetano La Manna | | |
| Parma | | | Azienda Ospedaliero-Universitaria di Parma | | | Umberto Maggiore | | |
| Pisa | | | Azienda Ospedaliero-Universitaria di Pisa Ospedale Cisanello | | | Ugo Boggi/Fabio Vistoli | | |
| Roma UCSC | | | Fondazione Policlinico Universitario A. Gemelli IRCCS | | | Jacopo Romagnoli/Giuseppe Grandaliano | | |
| Bari | | | Azienda Ospedaliero-Universitaria Policlinico di Bari | | | Loreto Gesualdo | | |
|  | | |  | | |  | | |
| **France** | | | | | | | | |
| **Centre** | | | **Hospital** | | | **Local Lead** | | |
| APHP - Paris | | | Necker Hospital | | | Pr Christophe Legendre | | |
| APHP - Paris | | | Pitié Salpêtrière Hospital | | | Pr Benoit Barrou | | |
| APHP - Paris | | | Saint-Louis Hospitals | | | Pr Carmen Lefaucheur | | |
| CHU Bordeaux | | | Pellegrin hospital | | | Pr Lionel Couzi | | |
| CHU Toulouse | | | Rangueil Hospital | | | Pr Nassim Kamar | | |
| CHU de Nantes | | | Hôtel Dieu | | | Pr Gilles Blancho | | |
| CHU Lyon | | | Edouard Herriot hospital | | | Pr Olivier Thaunat | | |
| CHU Montpellier | | | Lapeyronie Hospital | | | Pr Moglie Le Quintrec | | |
| CHU Lille | | | Huriez Hospital | | | Pr Marc Hazzan | | |
| CHU Tours | | | Trousseau hospital | | | Pr Matthias Büchler | | |
|  | |  | | |  | | |
| **Germany** | | | | | | | |
| **Centre** | | **Hospital** | | | **Local Lead** | | |
| Berlin | | Charité | | | Prof Klemens Budde | | |
| Mainz | | UK Mainz | | | Prof Martina Koch | | |
| Hannover | | MHH Hannover | | | Dr Nikolas Richter | | |
| Münster | | UKM Muenster | | | Prof Andreas Pascher | | |
|  | |  | | |  | | |
| **Spain** | | | | | | | |
| **Centre** | | **Hospital** | | | **Local Lead** | | |
| Santander | | Hospital de Santander | | | Daniel Casanova, Juan Carlos Ruiz | | |
| Madrid | | Hospital Universitario 12 de Octubre | | | Julio Pascual | | |
| Barcelona | | Bellvitge Hospital | | | Josep M. Cruzado | | |
| Barcelona | | Hospital Universitario Vall d'Hebron | | | Oriol Bestard | | |
| Zaragoza | |  | | | A Gutierrez | | |
| Sevilla | | Hospital Universitario Virgen Macarena | | | Rocio Molas Flores | | |
| Barcelona | | Fundació Puigvert | | | Lluis Guirado | | |
| Basque Country | |  | | | Francisco Gainza de los Rios | | |
| Barcelona | | Hospital Clinic | | | Fritz Diekmann | | |
| Sevilla | | Hospital Universitario Virgen Macarena | | | Mercedes Salgueira | | |
| Barcelona | | Hospital del Mar | | | Marta Crespo Barrio | | |
| Malaga | | Hospital Regional Universitario de Málaga | | | Domingo Hernandez | | |
|  | |  | | |  | | |

**Table S2.** COREQ Checklist

Consolidated Criteria for Reporting Qualitative Research (COREQ): 32-item checklist for focus groups. Adapted to reflect the complementary role of the qualitative component within the mixed-methods study design.

Reference: Tong A, Sainsbury P, Craig J. Consolidated criteria for reporting qualitative research (COREQ): a 32-item checklist for interviews and focus groups. Int J Qual Health Care. 2007;19(6):349–357.

| **No.** | **Item** | **Description** | **Reported in Section** | **Comment** |
| --- | --- | --- | --- | --- |
| ***Domain 1: Research team and reflexivity*** | | | | |
| ***Personal characteristics*** | | | | |
| 1 | **Interviewer/ facilitator** | Which author/s conducted the interview or focus group? | Methods, Qualitative Data Collection | Focus groups were moderated by a member of the research team with clinical and transplantation expertise. |
| 2 | **Credentials** | What were the researcher’s credentials? E.g. PhD, MD | Methods, Qualitative Data Collection | Clinician-researcher with transplantation expertise. Full credentials listed in author affiliations. |
| 3 | **Occupation** | What was their occupation at the time of the study? | Methods, Qualitative Data Collection | Academic researcher and clinician affiliated with the London School of Economics and collaborating transplant centres. |
| 4 | **Gender** | Was the researcher male or female? | N/A | Not reported. The moderator’s gender was not considered to influence the structured, domain-based focus group discussions. |
| 5 | **Experience and training** | What experience or training did the researcher have? | Methods, Qualitative Data Collection | Clinical and transplantation expertise, as stated in the manuscript. |
| ***Relationship with participants*** | | | | |
| 6 | **Relationship established** | Was a relationship established prior to study commencement? | Methods, Qualitative Data Collection | The moderator was not involved in participants’ clinical care. No prior relationship was established with participants. |
| 7 | **Participant knowledge of the interviewer** | What did the participants know about the researcher? E.g. personal goals, reasons for doing the research | Methods | Participants were informed of the study’s purpose (to identify best practices in kidney transplantation) and the moderator’s research role. |
| 8 | **Interviewer characteristics** | What characteristics were reported about the interviewer/facilitator? E.g. bias, assumptions, reasons and interests in the research topic | N/A | Not explicitly reported. The qualitative component was designed as complementary to the quantitative survey within a mixed-methods framework, reducing reliance on individual interviewer reflexivity. |
| ***Domain 2: Study design*** | | | | |
| ***Theoretical framework*** | | | | |
| 9 | **Methodological orientation and theory** | What methodological orientation was stated to underpin the study? E.g. grounded theory, discourse analysis, ethnography, phenomenology, content analysis | Methods, Qualitative Analysis | Thematic analysis within a mixed-methods design. An initial analytic framework was developed deductively based on the four study domains. |
| ***Participant selection*** | | | | |
| 10 | **Sampling** | How were participants selected? E.g. purposive, convenience, consecutive, snowball | Methods, Qualitative Data Collection | Purposive sampling to ensure representation across stakeholder groups and transplant centres. |
| 11 | **Method of approach** | How were participants approached? E.g. face-to-face, telephone, mail, email | Methods, Qualitative Data Collection | Recruitment was facilitated by designated local leads at each centre, who identified and invited eligible participants. |
| 12 | **Sample size** | How many participants were in the study? | Results / Supplementary Material | There were 17 participants in the Italian focus group. |
| 13 | **Non-participation** | How many people refused to participate or dropped out? Reasons? | N/A | Not systematically recorded. Participation was voluntary; formal tracking of refusals was not undertaken. Acknowledged as a limitation. |
| ***Setting*** | | | | |
| 14 | **Setting of data collection** | Where was the data collected? E.g. home, clinic, workplace | Methods, Qualitative Data Collection | Focus groups were conducted online. |
| 15 | **Presence of non-participants** | Was anyone else present besides the participants and researchers? | N/A | Not reported. Online focus groups were moderated by the research team; no non-participants were routinely present. |
| 16 | **Description of sample** | What are the important characteristics of the sample? E.g. demographic data, date | Results / Supplementary Material | Participants included patients, living donors, nephrologists, transplant surgeons, transplant coordinators, and hospital administrators. Demographic characteristics are provided in the Supplementary Material. |
| ***Data collection*** | | | | |
| 17 | **Interview guide** | Were questions, prompts, guides provided by the authors? Was it pilot tested? | Methods, Qualitative Data Collection; Supplementary Material | A semi-structured interview guide was developed based on preliminary survey findings and established guideline domains. The guide is provided in the Supplementary Material. |
| 18 | **Repeat interviews** | Were repeat interviews carried out? If yes, how many? | N/A | No repeat interviews were conducted. |
| 19 | **Audio/visual recording** | Did the research use audio or visual recording to collect the data? | Methods, Qualitative Data Collection | Audio recordings were made and transcribed verbatim. |
| 20 | **Field notes** | Were field notes made during and/or after the interview or focus group? | N/A | Not reported. Analysis relied on verbatim transcripts rather than field notes. |
| 21 | **Duration** | What was the duration of the interviews or focus group? | Methods, Qualitative Data Collection | Approximately 60–90 minutes per focus group. |
| 22 | **Data saturation** | Was data saturation discussed? | N/A | Not applicable. The qualitative component was designed to contextualise and complement quantitative survey findings within a mixed-methods framework, rather than to generate standalone qualitative theory. Data saturation was therefore not a design objective. |
| 23 | **Transcripts returned** | Were transcripts returned to participants for comment and/or correction? | N/A | Transcripts were not returned to participants. The qualitative analysis served a complementary, interpretive role within the mixed-methods design. |
| ***Domain 3: Analysis and findings*** | | | | |
| ***Data analysis*** | | | | |
| 24 | **Number of data coders** | How many data coders coded the data? | Methods, Qualitative Analysis | Three independent reviewers (SB, SM, VP). |
| 25 | **Description of the coding tree** | Did authors provide a description of the coding tree? | Methods, Qualitative Analysis | An initial analytic framework was developed deductively based on the four study domains and iteratively refined through transcript review. A formal coding tree was not presented, consistent with the complementary role of the qualitative component. |
| 26 | **Derivation of themes** | Were themes identified in advance or derived from the data? | Methods, Qualitative Analysis | Themes were developed deductively from the four study domains (aligned with the survey sections) and iteratively refined through transcript review. |
| 27 | **Software** | What software, if applicable, was used to manage the data? | N/A | No dedicated qualitative software was used; analysis was conducted manually |
| 28 | **Participant checking** | Did participants provide feedback on the findings? | N/A | Participant checking was not conducted. Findings were validated through triangulation with quantitative survey data and consensus among three independent reviewers. |
| ***Reporting*** | | | | |
| 29 | **Quotations presented** | Were participant quotations presented to illustrate the themes/findings? Was each quotation identified? | Supplementary Material | Illustrative quotations are presented in Supplementary Table S3, mapped to key thematic domains and identified by stakeholder role and country. |
| 30 | **Data and findings consistent** | Was there consistency between the data presented and the findings? | Results; Discussion | Yes. Qualitative themes were mapped against quantitative survey findings to ensure consistency and complementarity. |
| 31 | **Clarity of major themes** | Were major themes clearly presented in the findings? | Results | Yes. Major themes are presented under nine thematic headings aligned with the four study domains and ten best practice recommendations. |
| 32 | **Clarity of minor themes** | Is there a description of diverse cases or discussion of minor themes? | Results; Discussion | Country-specific variations and minority perspectives are discussed where relevant. The uneven representation of certain stakeholder groups is acknowledged as a limitation. |

**Table S3.** Illustrative focus group quotations mapped to key thematic domains

Quotations are drawn from focus group discussions conducted in each of the four participating countries and are mapped to key thematic domains identified in the study. Quotations have been translated into English where necessary. Participants are identified by stakeholder role and country to preserve anonymity.

| **Domain** | **Theme** | **Illustrative quotation** | **Role** | **Country** |
| --- | --- | --- | --- | --- |
| CKD Monitoring | Early diagnosis challenges | *“The biggest hurdle is to identify patients with chronic kidney disease […] The family doctors don’t always have an eye on it. And often a lot of time goes by before it is recognised that there is a problem.”* | Nephrologist | Germany |
| CKD Monitoring | Variability in monitoring practices | *“Monitoring often depends on individual initiative; rural patients suffer the most from gaps in follow-up.”* | Nephrologist | Germany |
| CKD Monitoring | Patient pathways and information | *“Patients often arrive late and confused, as their care pathway is too frequently left to the experience and judgment of local nephrologists. […] It’s important for nephrologists to empower patients to make informed, autonomous choices about dialysis or transplantation.”* | Nephrologist | Italy |
| Living Donation | Late introduction of living donor options | *“When dialysis was proposed, I researched and discovered the option of living donation, which no one had mentioned to us. Once we communicated our intention to the centre, we received comprehensive information about the process and clarifications about the risks.”* | Living donor | Italy |
| Living Donation | Regional fragmentation in donor awareness | *“The reality is highly fragmented. There are areas where certain options, like living donation, are not discussed, and patients come to larger centres saying no one had ever informed them about it.”* | Transplant coordinator | Italy |
| Donor Communication | Importance of transparent communication | *“The expertise of the hospital doctors dispelled my doubts and fears. Clear communication reassures donors and helps them make a definitive decision.”* | Living donor | Italy |
| Post-transplant Care | Donor follow-up concerns | *“In my experience, my daughter received consistent care even after the transplant, with periodic check-ups and close monitoring when issues arose. However, as her donor, I feel somewhat neglected.”* | Living donor | Italy |
| QoL Assessment | Absence of standardised QoL tools | *“I routinely ask patients how they feel after transplantation and about their QoL, but I don’t record this information as I do clinical data. As a result, I have a subjective sense of post-transplant QoL but no objective data.”* | Nephrologist | Italy |
| QoL Assessment | Need for systematic measurement | *“Measuring QoL is crucial, particularly to encourage more donors. […] However, QoL assessment often takes a backseat in daily practice due to a lack of tools and time.”* | Transplant coordinator | Italy |
| Post-transplant Support | Psychological support needs | *Participants expressed a need for better psychological support and peer mentoring, with many describing a desire for stronger community networks to connect recipients and donors.* | Multiple participants | France |
| Governance & Funding | Financial constraints on innovation | *“Current policies often fail to address the financial realities faced by transplant centres, leading to suboptimal patient care.”* | Focus group participant | France |
| Governance & Funding | Reimbursement adequacy | *Participants felt the DRG system does not reflect the true cost of transplantation, particularly for pharmaceuticals and post-operative care, and expressed concern about the lack of financial incentives tied to patient outcomes.* | Multiple participants | France |

**Table S4.** Focus group participant characteristics by country: a) Germany, b) Italy, c) France, d) Spain

a)

|  | **Session 1 (12 November 2024** |
| --- | --- |
| **Total participants** | 3 |
| ***By stakeholder role*** | |
| Transplant surgeon | 1 |
| Transplant coordinator | 0 |
| Nephrologist | 1 |
| Living donor | 0 |
| Recipient (patient) | 1 |
| Hospital administrator | 0 |
| ***By transplant centre*** | |
| University Hospital Münster, Germany | 3 |

**Moderators: Dr. Philipp Houben, Transplant Surgeon**

**Date of session: November 12, 2024**

b)

|  | **Session 1 (19 July 2023)** | **Session 2 (26 July 2023)** | **Total** |
| --- | --- | --- | --- |
| **Total participants** | **9** | **8** | **17** |
| ***By stakeholder role*** | | | |
| Transplant surgeon | 2 | 2 | 4 |
| Transplant coordinator | 2 | 1 | 3 |
| Nephrologist | 1 | 2 | 3 |
| Living donor | 2 | 1 | 2 |
| Recipient (patient) | 1 | 1 | 2 |
| Hospital administrator | 1 | 1 | 3 |
| ***By transplant centre*** | | | |
| Gemelli – Rome | 5 | 2 | 7 |
| Padova | 1 | 0 | 1 |
| Bologna | 0 | 2 | 2 |
| Milano – Policlinico | 0 | 1 | 1 |
| Milano – Niguarda | 3 | 0 | 3 |
| Bari | 0 | 3 | 3 |

c)

|  | **Session 1 (21 November 2024)** | **Session 2 (22 November 2024)** | **Total** |
| --- | --- | --- | --- |
| **Total participants** | 6 | 5 | 11 |
| ***By stakeholder role*** | | | |
| Transplant surgeon | 1 | 1 | 2 |
| Transplant coordinator | 1 | 1 | 2 |
| Nephrologist | 2 | 2 | 4 |
| Living donor | 0 | 0 | 0 |
| Recipient (patient) | 1 | 0 | 1 |
| Hospital administrator | 1 | 1 | 2 |
| ***By transplant centre*** | | | |
| CHU de Nantes | 2 | 0 | 2 |
| CHU de Bordeaux | 2 | 0 | 2 |
| Hôpital Saint-Louis APHP Paris | 2 | 3 | 5 |
| Hôpital Necker APHP Paris | 0 | 1 | 1 |

d)

|  | **Session 1 (23 September 2023)** | **Session 2 (23 October 2023)** | **Total** |
| --- | --- | --- | --- |
| **Total participants** | 8 | 8 | 16 |
| ***By stakeholder role*** | | | |
| Transplant surgeon | 1 | 1 | 2 |
| Transplant coordinator | 1 | 1 | 2 |
| Nephrologist | 3 | 3 | 6 |
| Living donor | 1 | 1 | 2 |
| Recipient (patient) | 1 | 1 | 2 |
| Hospital administrator | 1 | 1 | 2 |
| ***By transplant centre*** | | | |
| Hospital Universitario Cruces | 7 | 7 | 14 |
| Hospital Universitario Marques de Valdecilla | 1 | 1 | 2 |
| Hospital Del Mar- Barcelona | 0 | 0 | 0 |
| Hospital Vall de Hebron -Barcelona | 0 | 0 | 0 |
| Hospital 12 de Octubre -Madrid | 0 | 0 | 0 |

**Table S5. Focus Group Moderator Instructions and Discussion Framework**

**Study:** Best Practices Along the Kidney Transplantation Clinical Journey
**Component:** Qualitative Focus Group Discussions
**Format:** Semi-structured; online; approximately 60–90 minutes
**Participants:** Transplant surgeons, nephrologists, transplant coordinators, hospital administrators, living donors, and transplant recipients

**Note:** This document represents the instructions and thematic framework provided to moderators prior to each focus group session. Moderators were not required to follow a formal script or pose all questions listed; rather, they were asked to ensure that discussion covered the four core thematic domains of the study, drawing on the example questions and prompts below as appropriate to guide and structure conversation.

**Opening instructions for moderators**

Moderators were asked to begin each session by welcoming participants warmly and introducing themselves and their role in the study. They were instructed to provide a brief, accessible overview of the project, explaining that the study aimed to identify best practices along the kidney transplantation clinical journey across four European countries, combining a large survey with focus group discussions to capture real-world perspectives from a range of stakeholders.

Moderators were asked to explain that the session would cover four broad themes: CKD monitoring and early management, kidney donation and transplantation, post-transplant recipient care, and healthcare governance and service organisation. Participants did not need to have expertise across all areas and were encouraged to contribute wherever they felt they had relevant experience or views.

Informed consent was to be established at the start of the session. Moderators were asked to explain that the session would be recorded and transcribed for research purposes only, that all contributions would be treated confidentially and reported anonymously, and that participation was entirely voluntary. Participants were informed that remaining on the call would be taken as confirmation of their consent to participate.

Moderators were asked to set a welcoming and open tone, emphasising that there were no right or wrong answers and that the research team was interested in honest, practical perspectives, including where current practice falls short. Participants were encouraged to build on each other's contributions and to raise issues they felt were important, even if not directly prompted.

**Domain 1: CKD Monitoring and Early Management**

*Suggested opening:* "To begin, I'd like to discuss how chronic kidney disease is identified and managed in your setting, particularly in the earlier stages."

**Example questions:**

1. How is CKD typically identified and monitored in your centre or region? Are there standardised protocols in place, or does practice vary?
2. In your experience, at what stage do patients tend to be referred to specialist nephrology care? Do you feel this timing is appropriate?
3. What barriers, if any, exist to early diagnosis or timely referral — for example, in terms of primary care capacity, patient awareness, or geographic access?
4. Are there differences in CKD monitoring or referral practices between urban and rural settings in your country? How significant do you feel these disparities are?

**Example follow-up prompts:**

- *"Can you give an example of a case where earlier detection would have made a difference?"*
- *"How well do you feel primary care colleagues are equipped to manage early CKD?"*
- *"What changes, if any, would most improve CKD monitoring in your setting?"*

**Domain 2: Kidney Donation and Transplantation**

*Suggested opening:* "I'd now like to move on to the donation and transplantation process itself — including both deceased and living donation."

**Example questions:**

1. How would you describe the current approach to donor identification and referral in your centre? What works well, and where do you see gaps?
2. How is living donation discussed with patients and their families in your practice? At what point in the patient journey does this conversation typically occur?
3. What do you feel are the main barriers to living donation in your setting — from the perspective of patients, donors, or clinicians?
4. Are there particular aspects of donor coordination — such as collaboration between teams, communication protocols, or resource availability — that you feel could be strengthened?

**Example follow-up prompts:**

- *"Are there cases where living donation was not discussed until relatively late? What drove that?"*
- *"How do patients typically find out about living donation as an option?"*
- *"What educational or informational resources are currently available to patients and potential donors?"*

**Domain 3: Post-Transplant Recipient Care**

*Suggested opening:* "The next area I'd like to explore is care after transplantation — both in the immediate post-operative period and longer term."

**Example questions:**

1. How is post-transplant follow-up structured at your centre? Who leads that care, and how frequently do patients attend?
2. In your experience, are there areas where post-transplant monitoring could be more consistent or better coordinated — for example, between transplant centres and local nephrologists?
3. How well are quality-of-life outcomes and psychological wellbeing currently addressed in post-transplant care? Are there gaps?
4. For living donors specifically: what follow-up support is available after donation? Do you feel this is adequate?

**Example follow-up prompts:**

- *"Are there patient groups who you feel are particularly underserved in post-transplant care?"*
- *"What role, if any, do patient-reported outcomes currently play in your follow-up assessments?"*
- *"Are there specific aspects of long-term care that you feel receive insufficient attention?"*

**Domain 4: Healthcare Governance and Service Management**

*Suggested opening:* "Finally, I'd like to discuss the wider governance and organisational context — how transplantation services are structured, resourced, and evaluated."

**Example questions:**

1. How would you describe the governance structure of transplantation services in your country or centre? Do you feel it supports effective, consistent care delivery?
2. What are the main resource or workforce challenges facing transplant services in your setting? How do these affect the care you are able to provide?
3. How are outcomes currently measured and monitored at your centre? Are these metrics meaningful and useful for quality improvement?
4. How would you characterise the reimbursement and funding model for transplantation in your country? Does it support innovation and equitable access to care?

**Example follow-up prompts:**

- *"Are there aspects of governance or coordination across centres that you feel could be streamlined?"*
- *"How do financial constraints affect decisions about patient care or service development in practice?"*
- *"Are there particular policies or structural changes you feel would have the greatest impact on improving care?"*

**Closing**

*"We are coming to the end of our discussion. Before we close, I'd like to give everyone an opportunity to raise anything we haven't yet covered."*

1. Is there anything else — related to any stage of the kidney transplantation pathway — that you feel is important for this study to capture?
2. If you could identify **one priority change** that would most improve kidney transplantation care in your setting, what would it be?

*"Thank you very much for your time and contributions. Your insights will be invaluable in informing the best practice recommendations that emerge from this study."*

**Notes for Moderators**

- The moderators were given the full survey questions and set of instructions and thematic guidance, not a prescriptive script. Moderators were asked to ensure that discussion addressed each of the four core domains, but were free to adapt the order, wording, and depth of exploration based on the flow of each session and the issues participants identified as most relevant.
- The example questions and prompts listed above are illustrative; moderators were not required to pose them verbatim or in full.
- Questions should be adapted as needed to reflect the specific role and experience of participants (e.g., clinical questions will be most relevant to healthcare professionals; questions on patient experience and information access will be most relevant to patients and living donors).
- The framework was developed on the basis of preliminary survey findings and established guideline domains (KDIGO, European Commission CD-P-TO, British Transplantation Society guidelines), and was applied consistently across all four participating countries to ensure thematic comparability.
- All sessions were conducted in the local language; moderators applied the framework accordingly.

### **Figure S1.** Ethics approval by country

### Italy


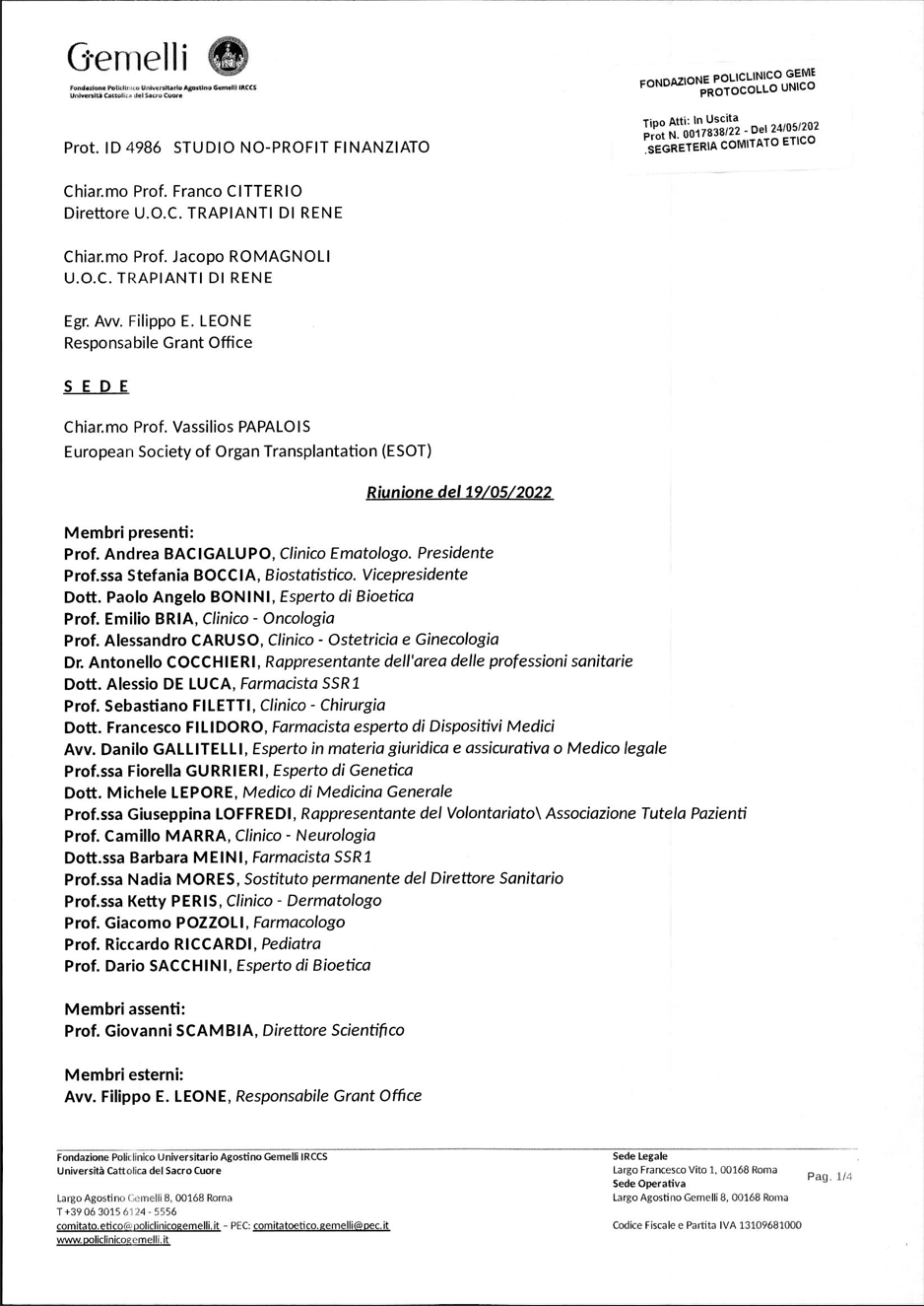


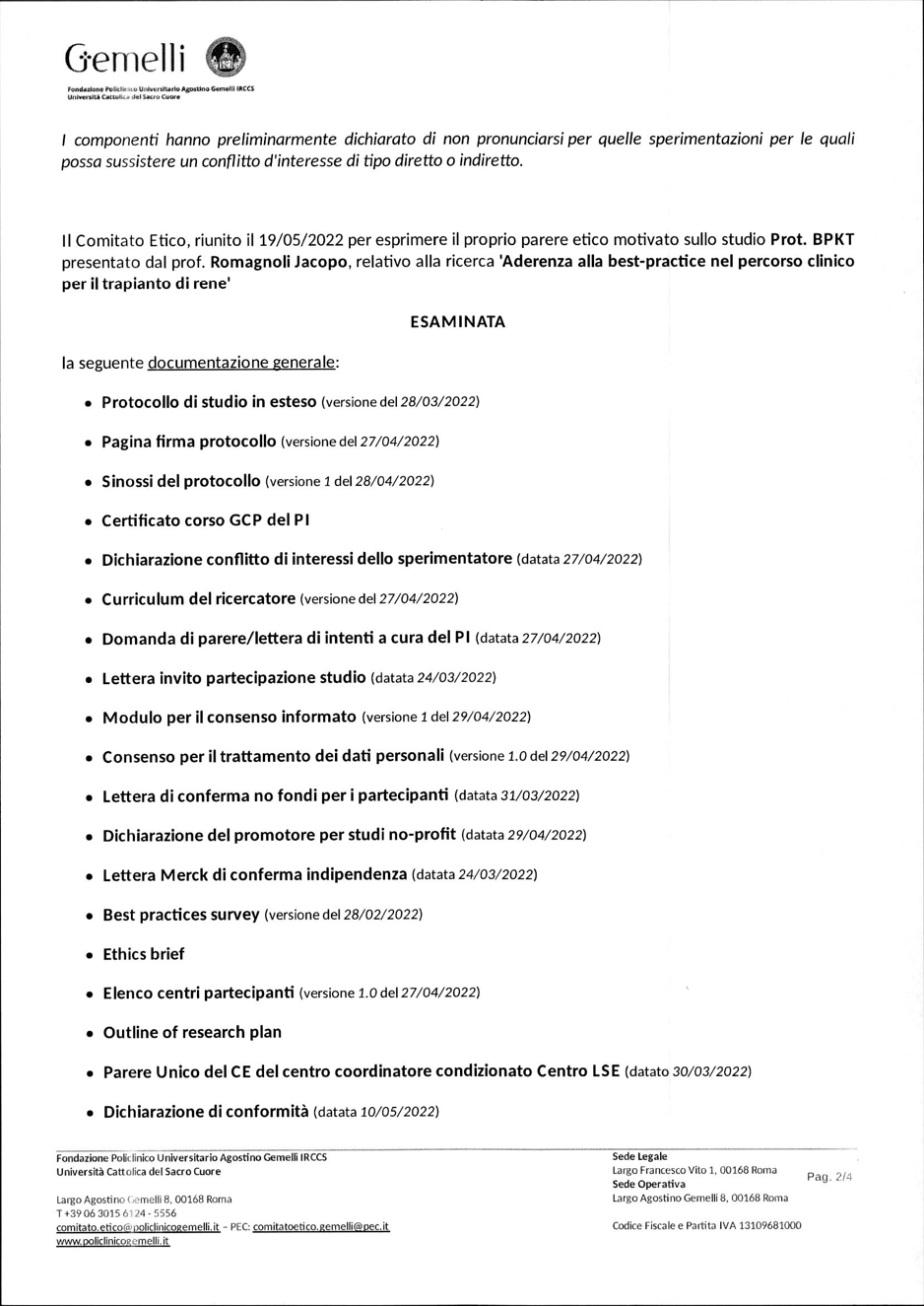


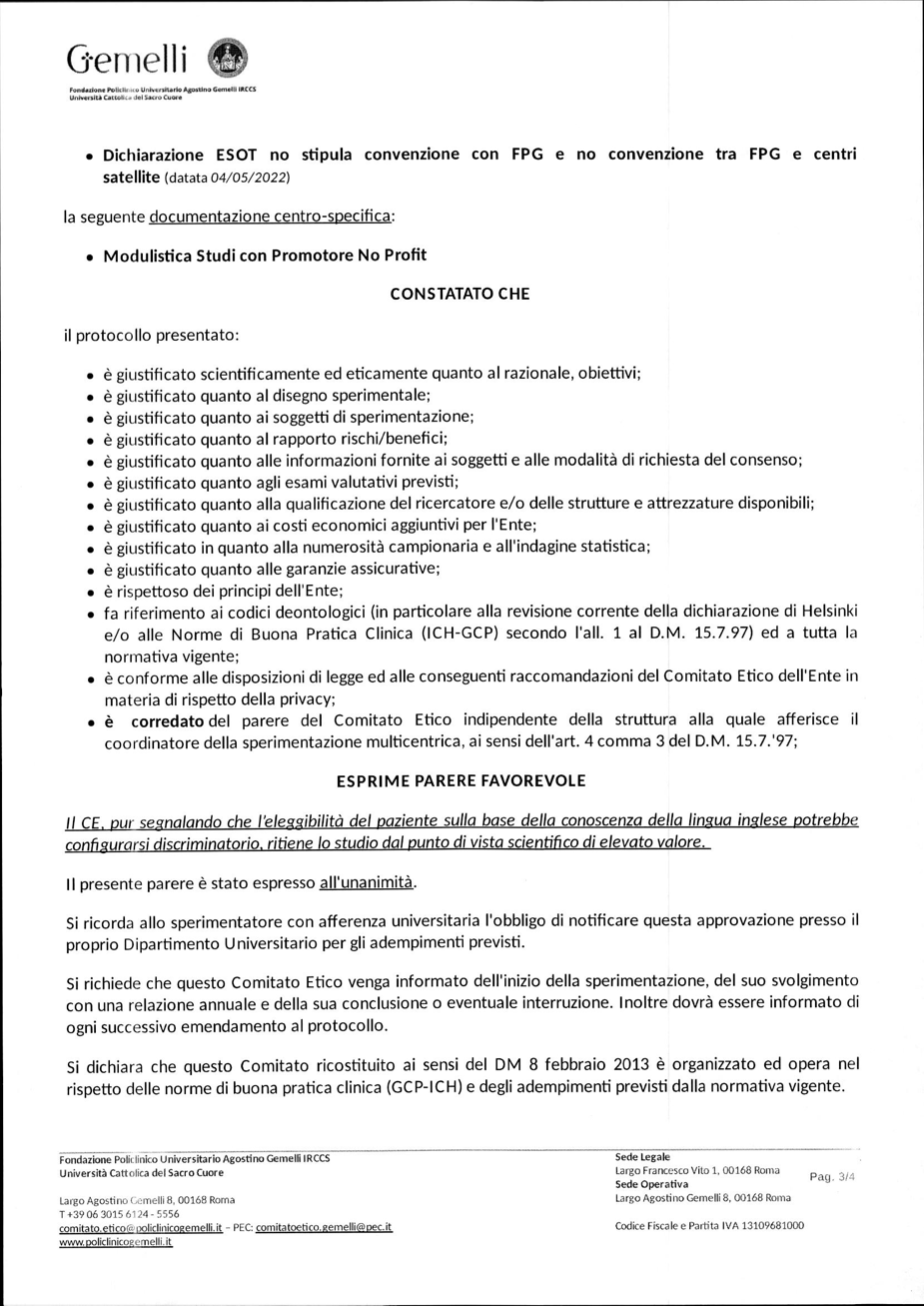


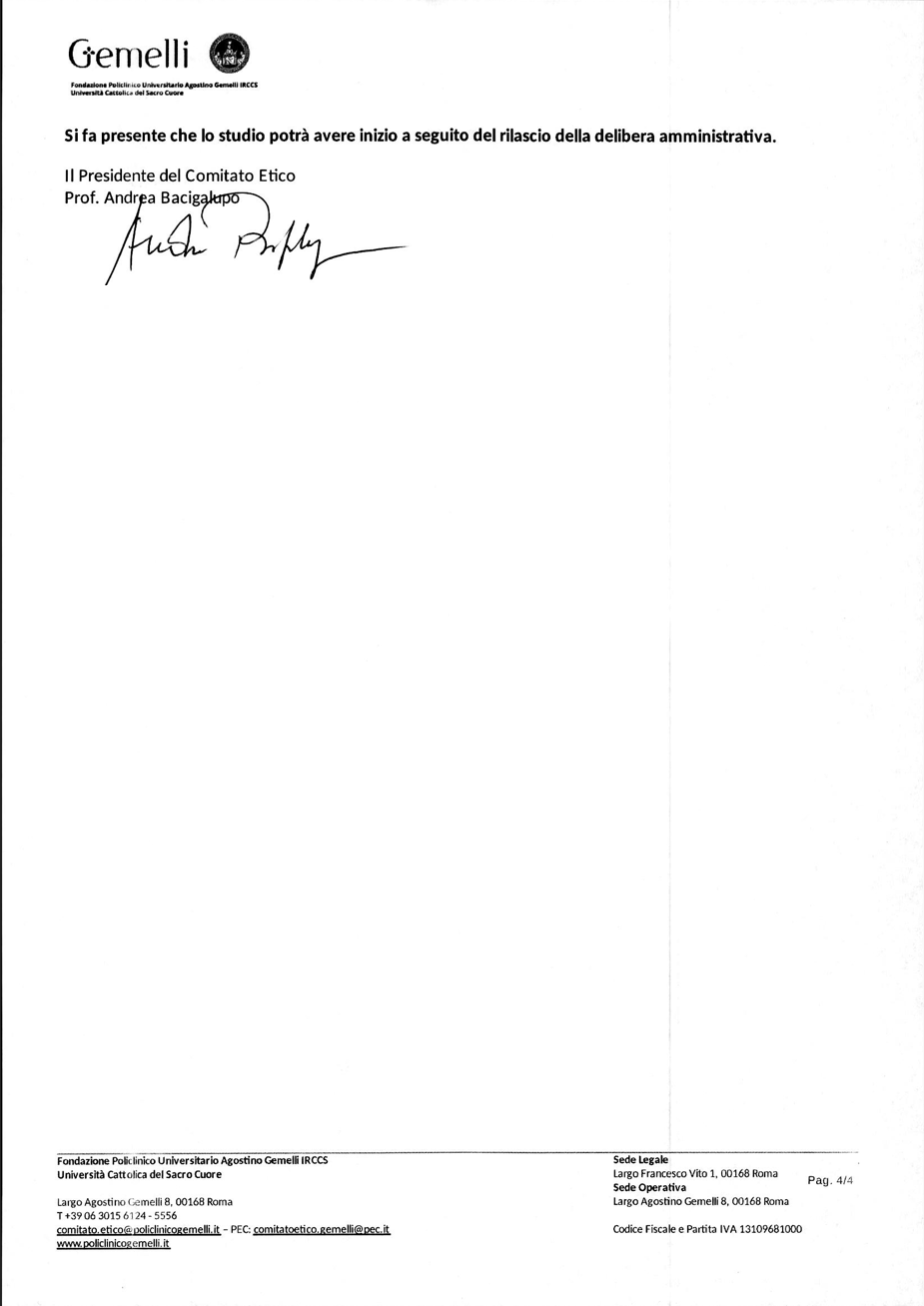


### France


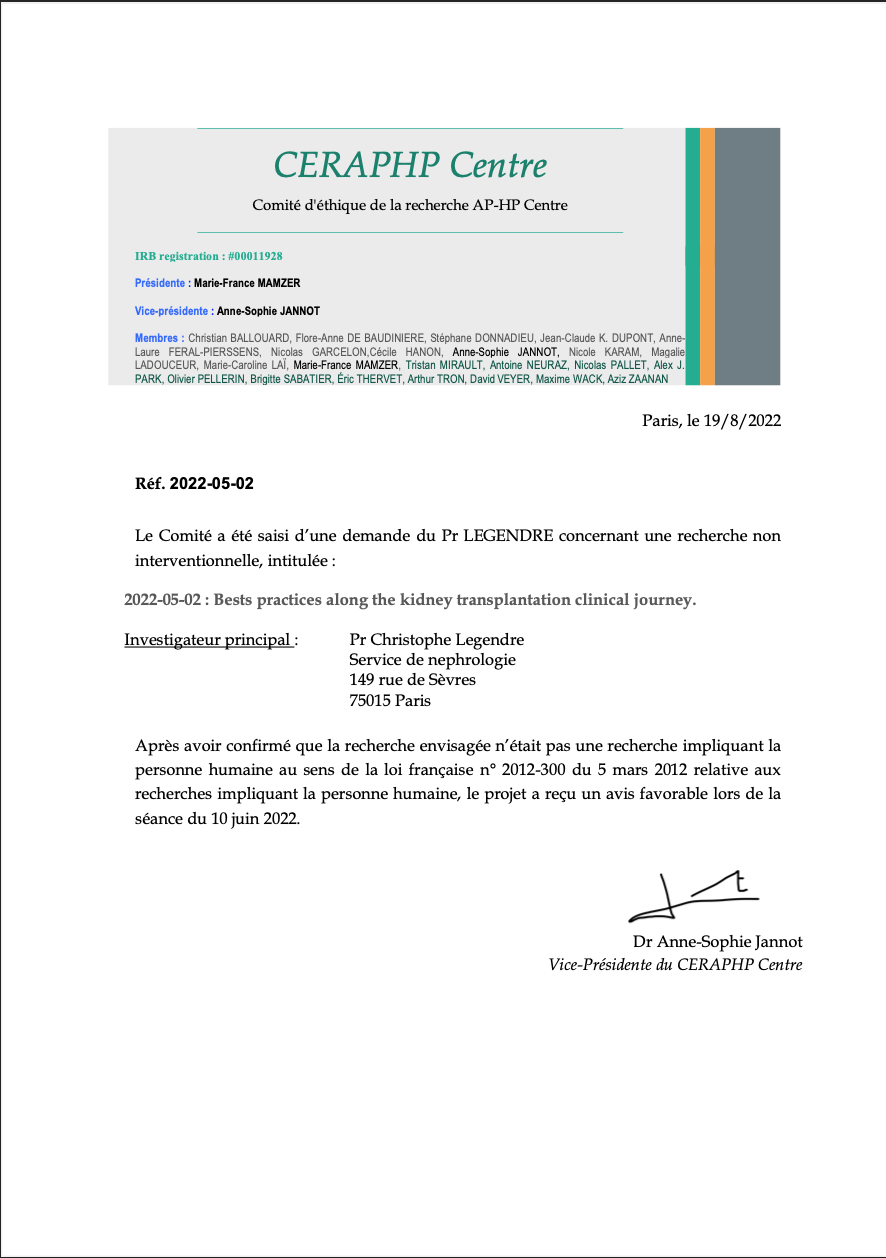


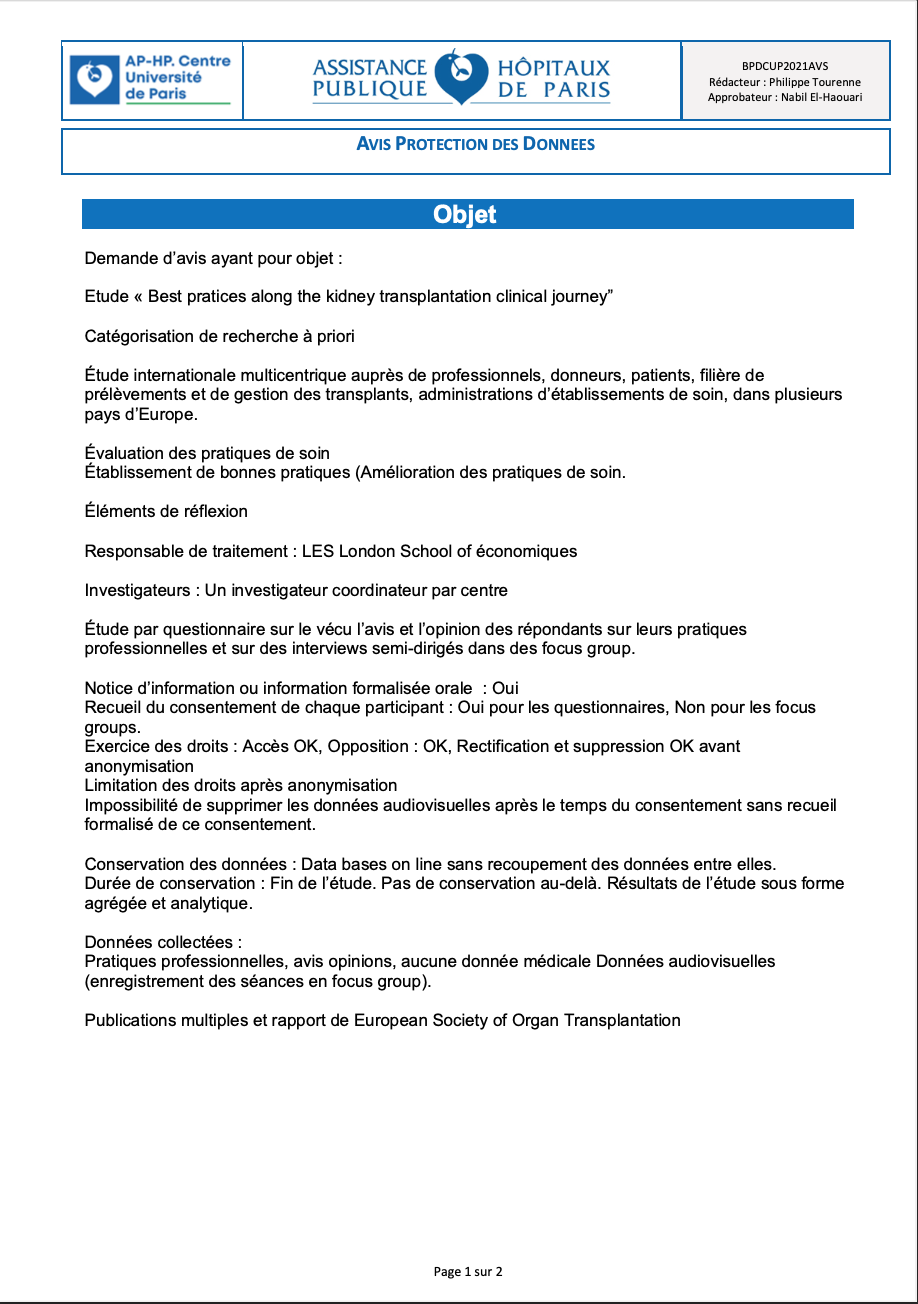


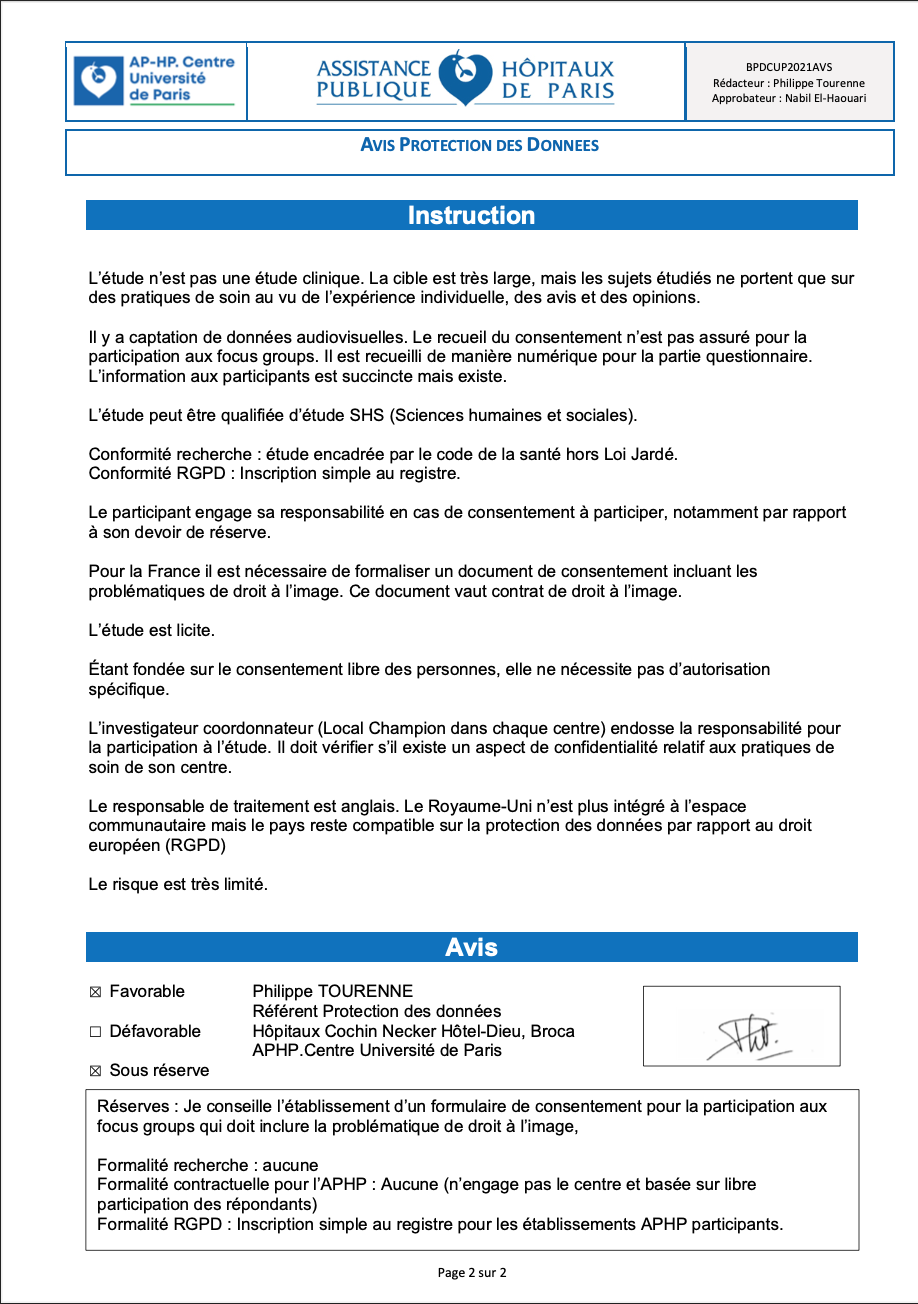


### London School of Economics - UK


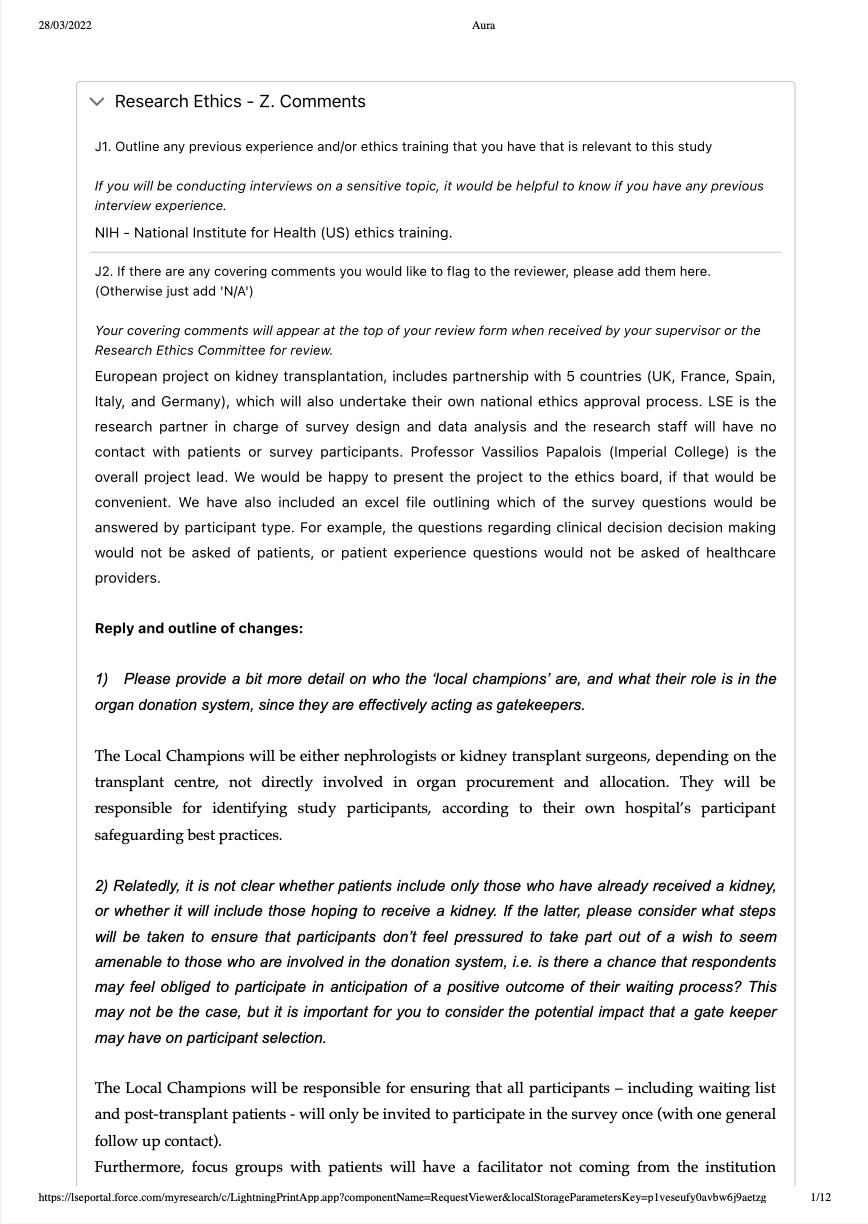


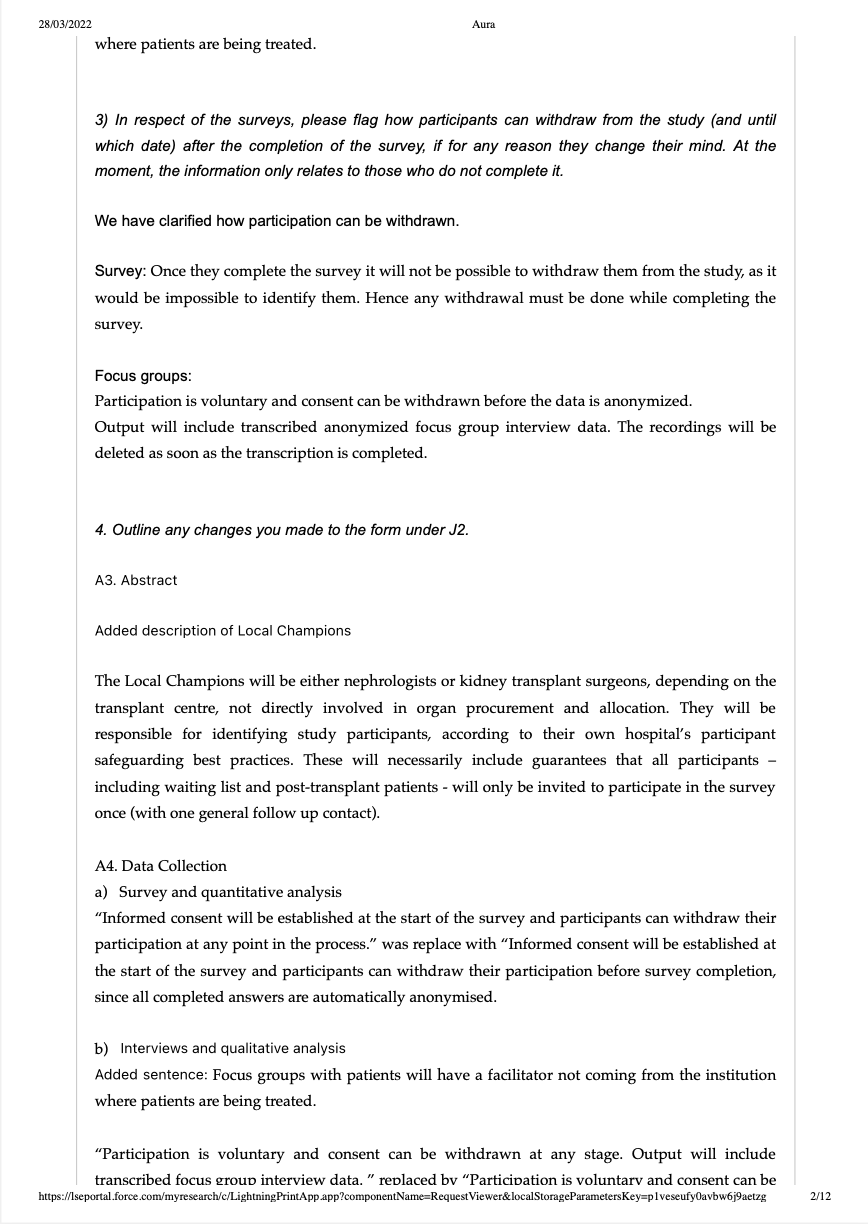


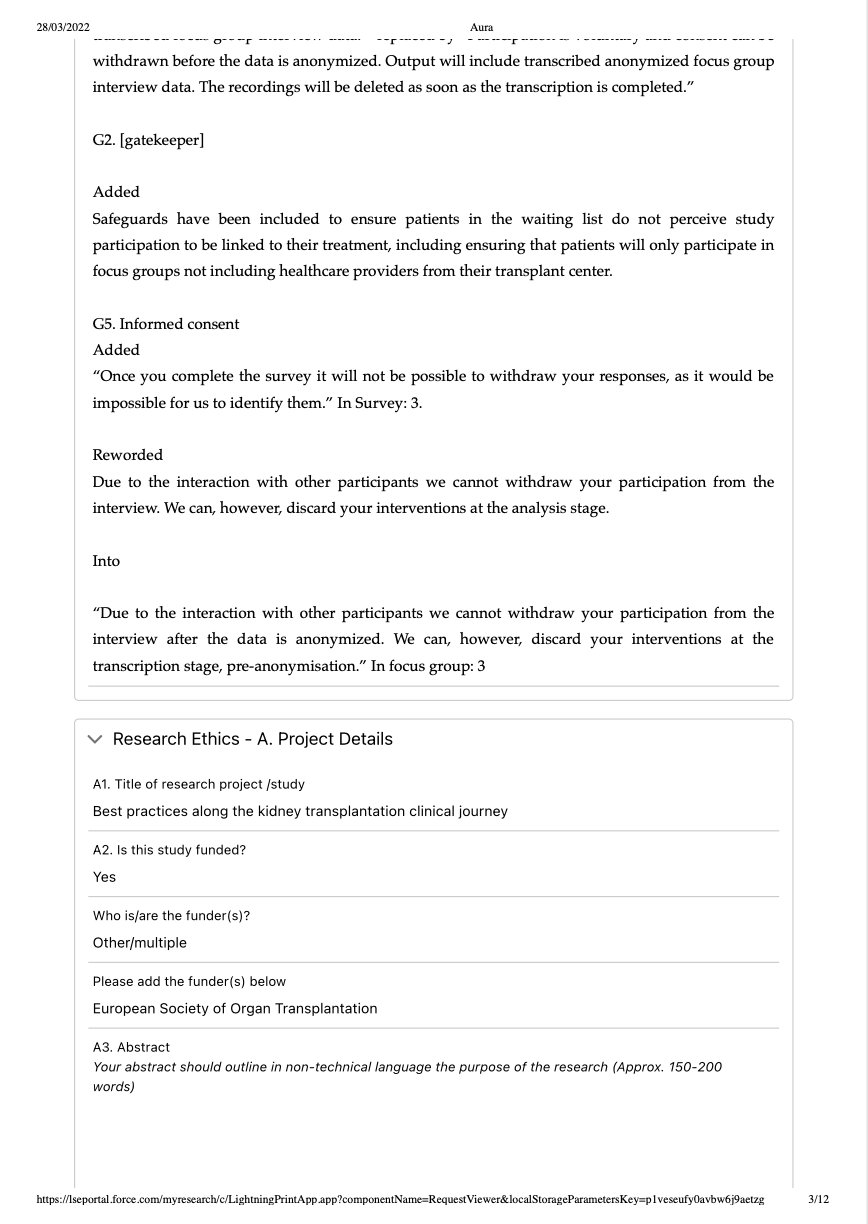


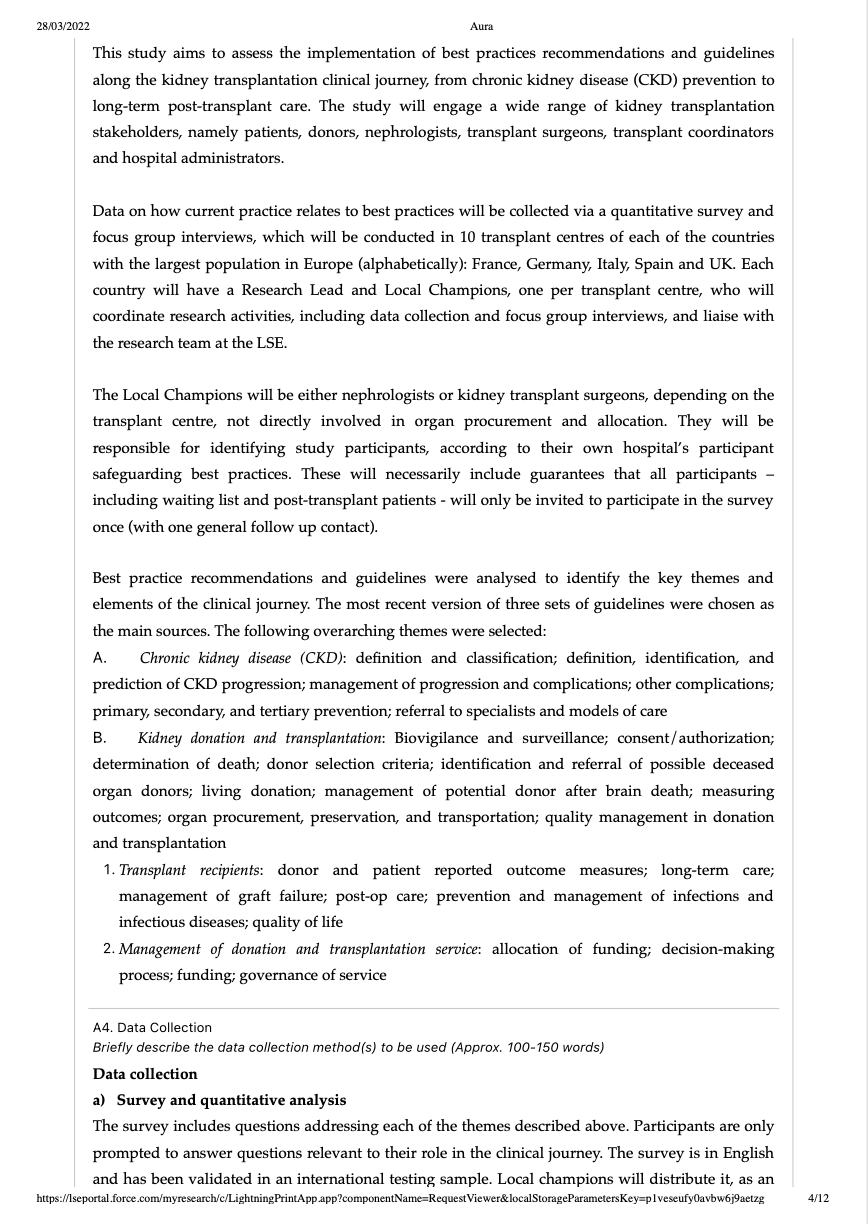


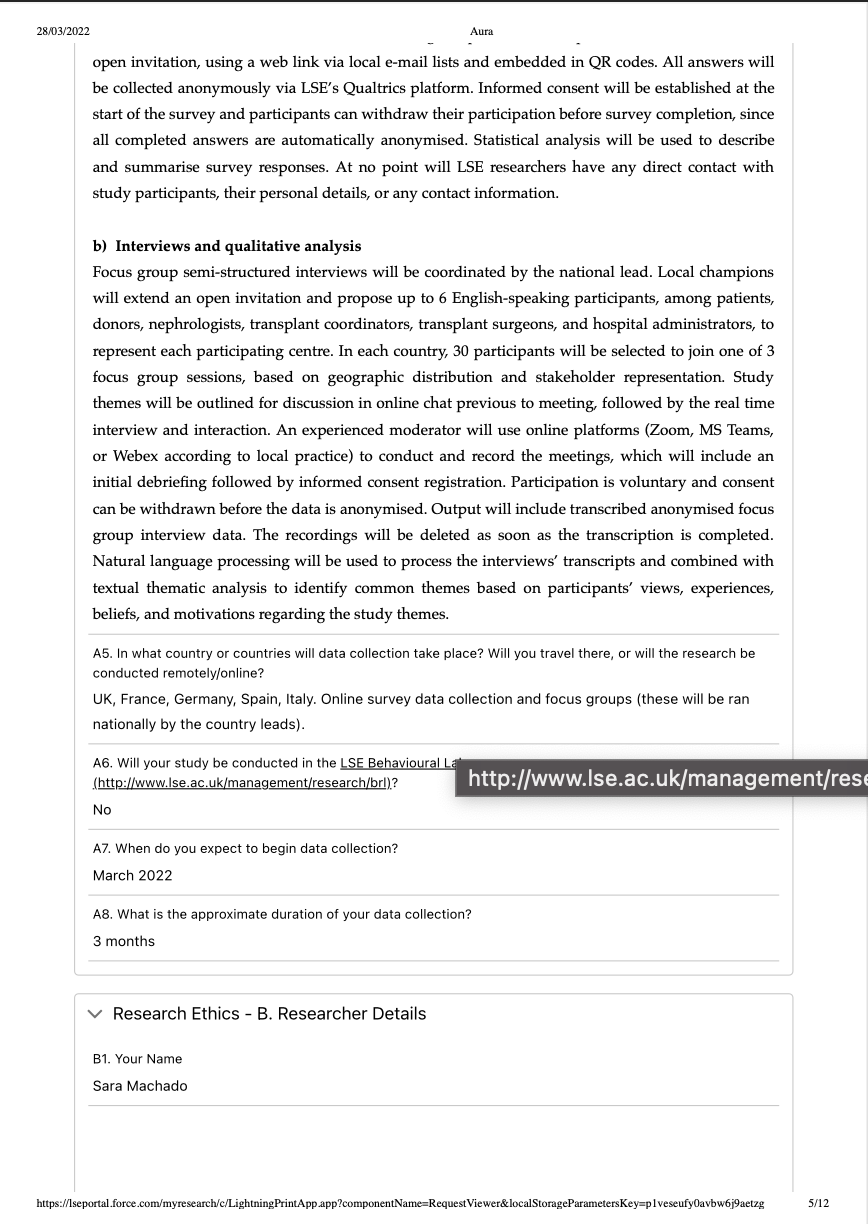


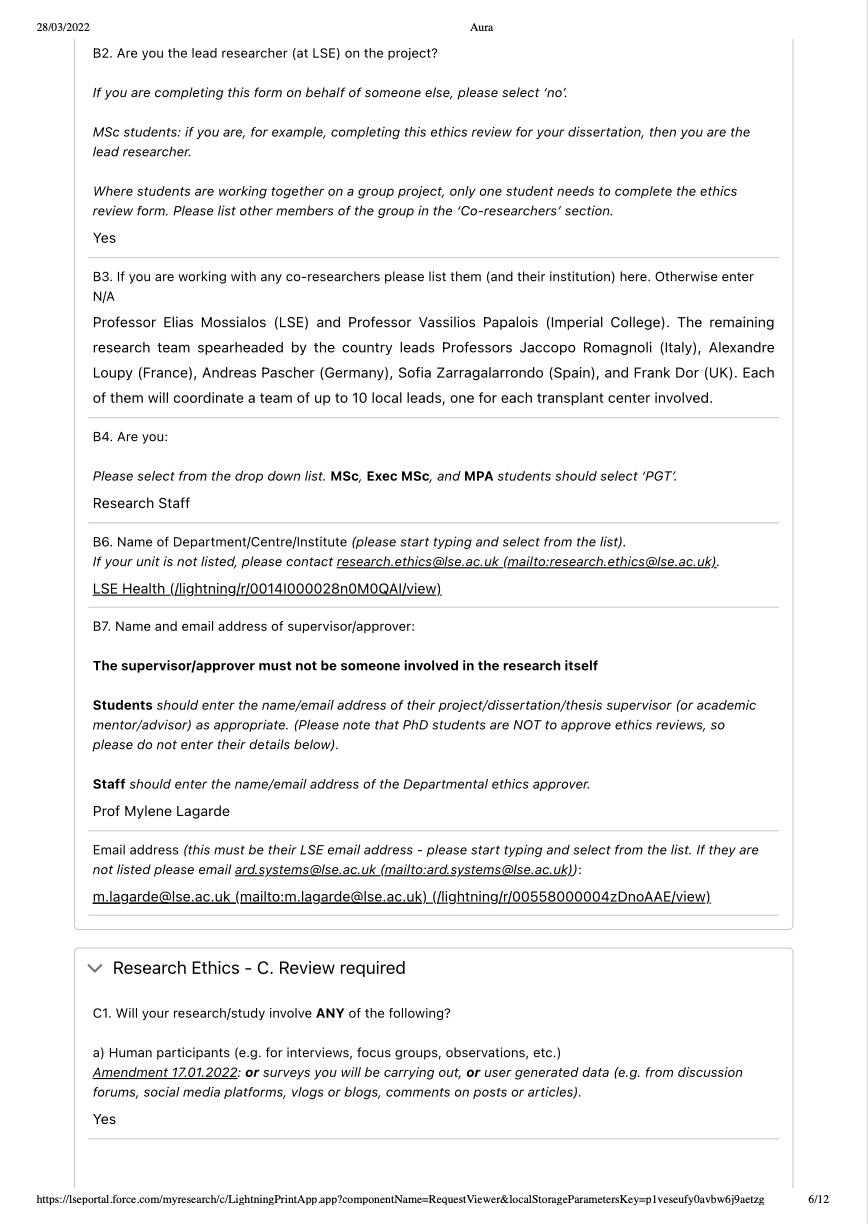


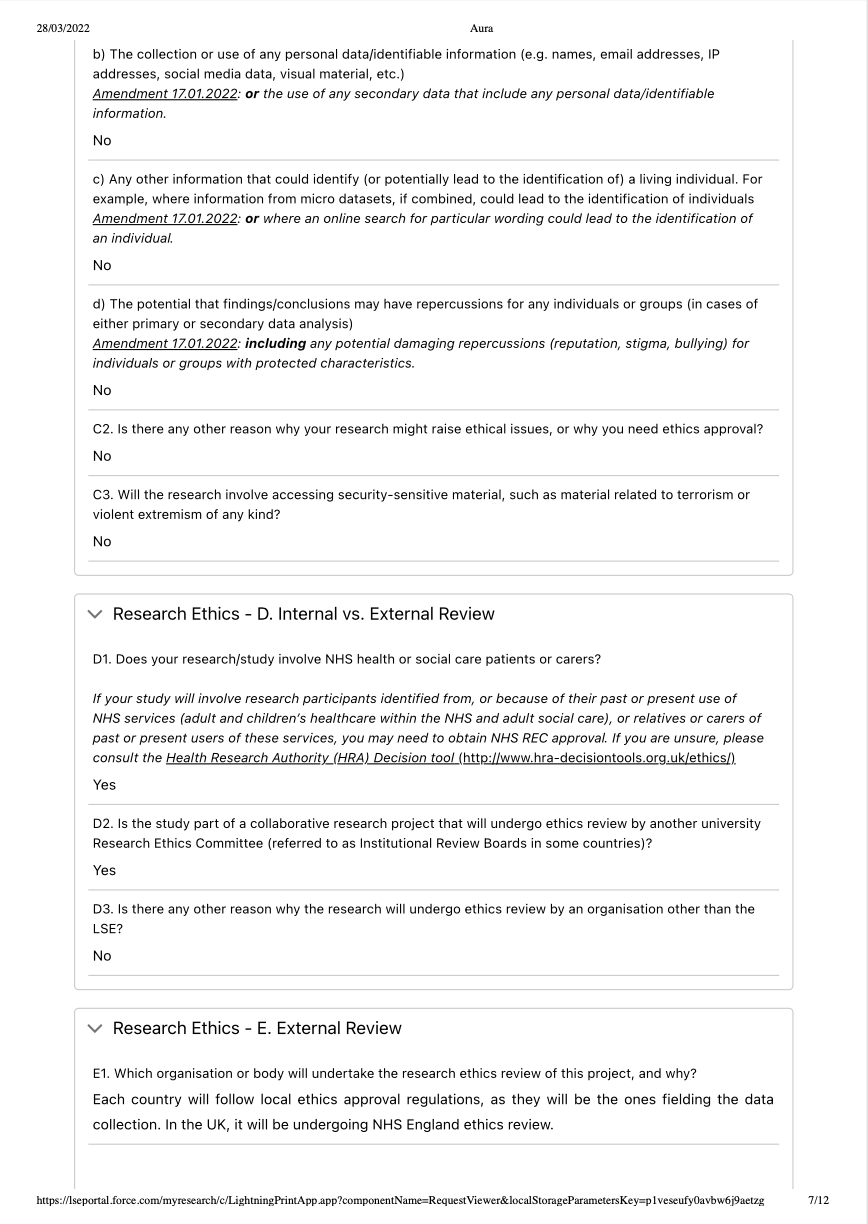


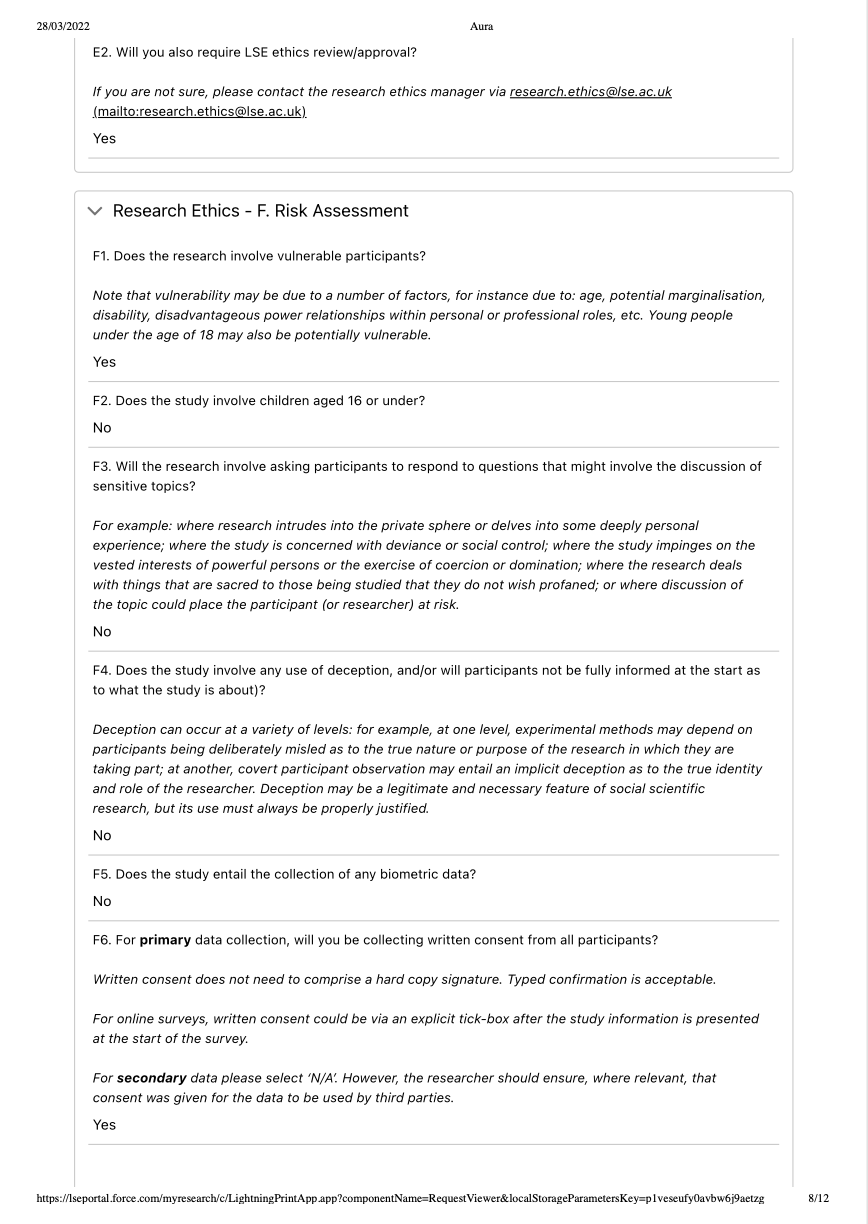


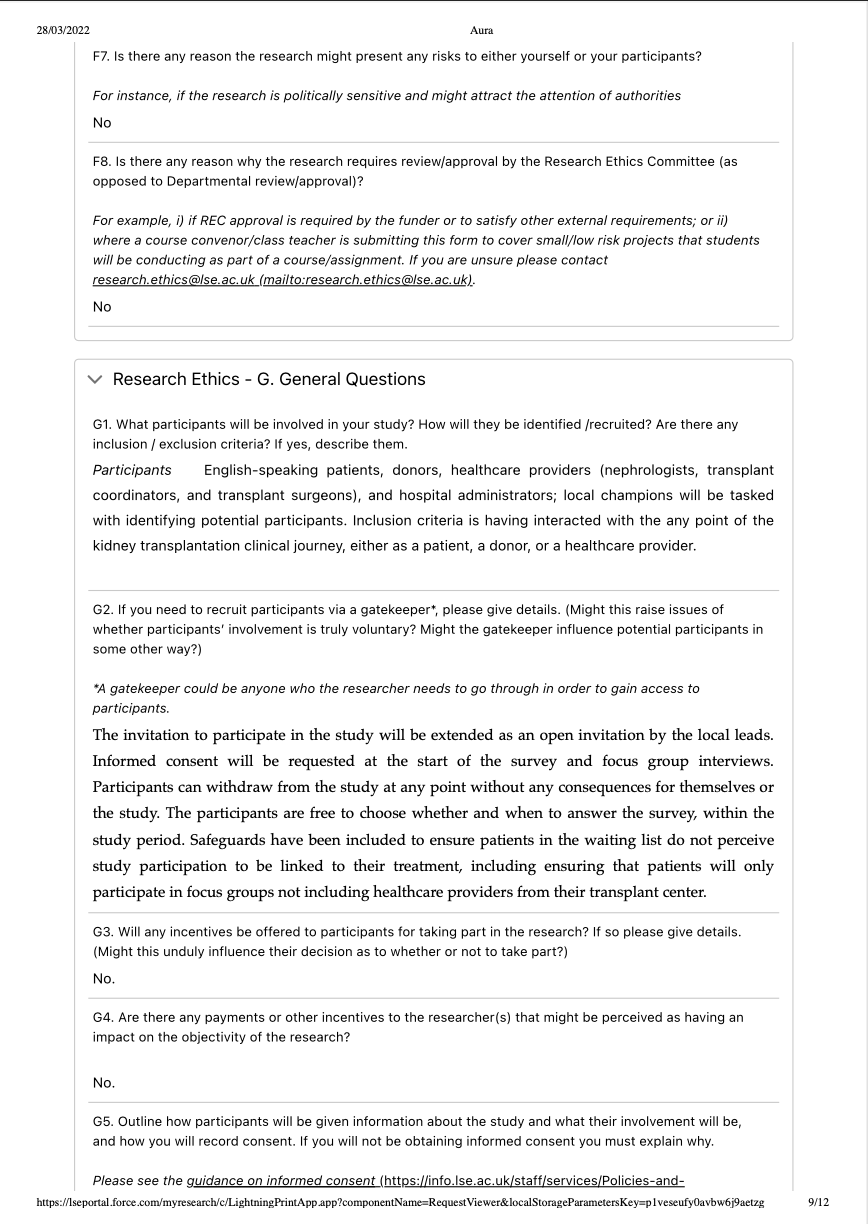


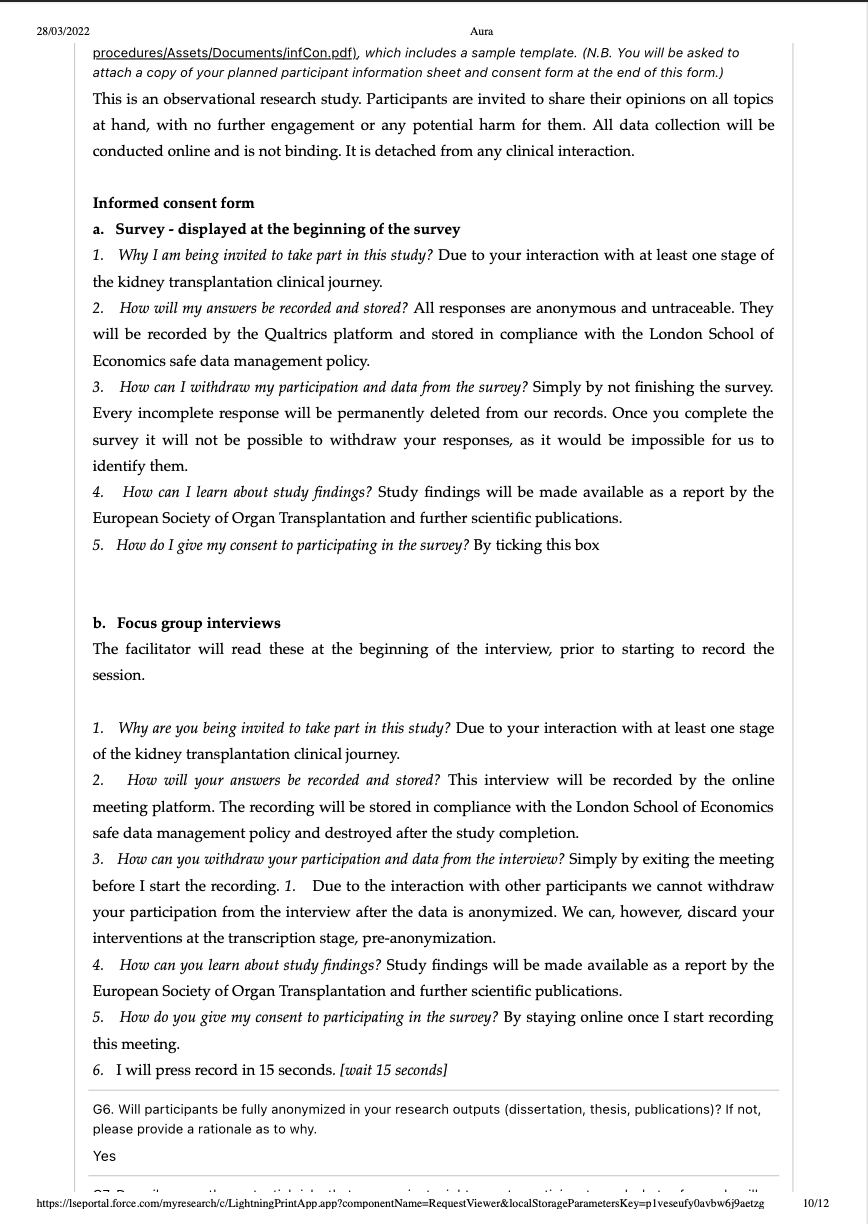


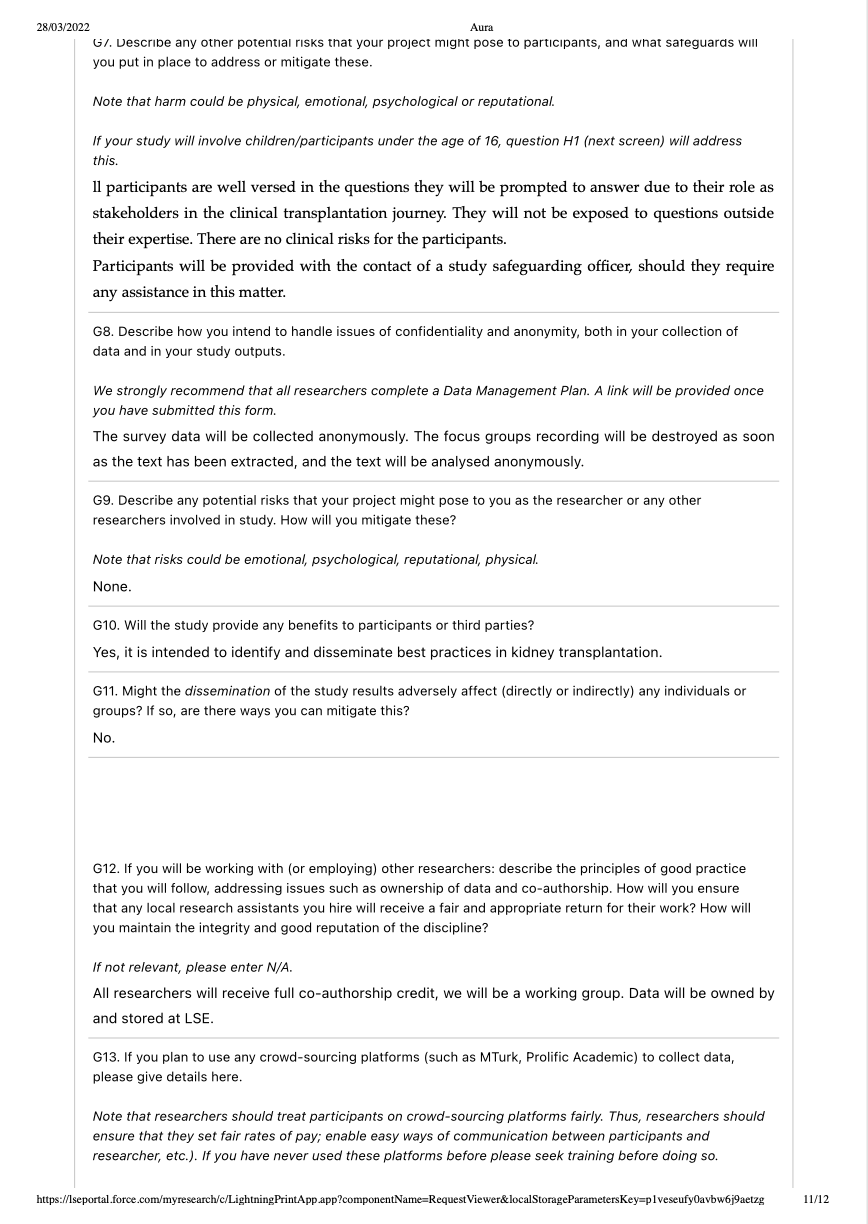


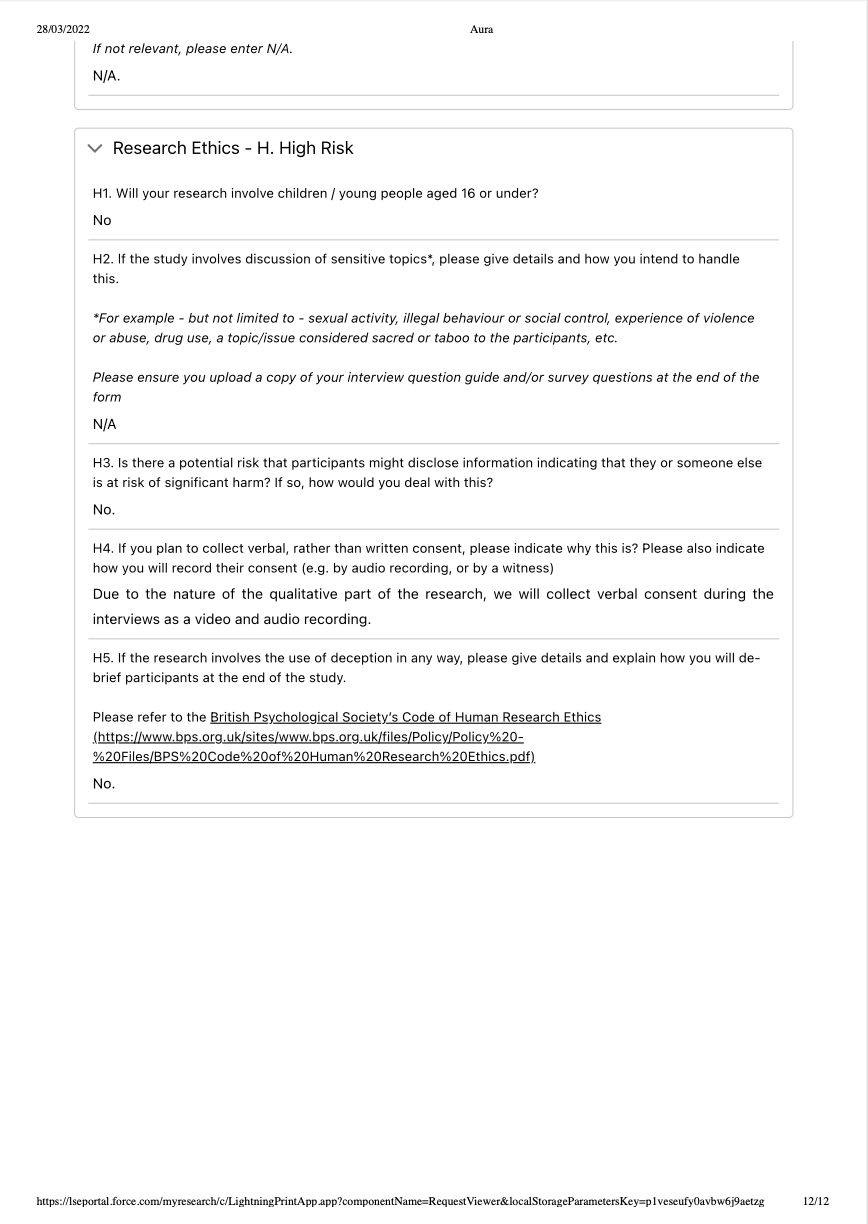


**Figure S2.** Survey Questions

## Chronic kidney disease

## Kidney donation and transplantation

## Transplant recipients

## Management of donation and transplantation service

**European Society of Organ Transplantation (ESOT) for Best Practices in Kidney Transplantation**

**France/Italy/Spain/Germany**

**Section 1 - Demographic Data**

**Table 1.** Demographic details of questionnaire respondents (n = 253).

| Demographic Details | n (%)/mean (± S.D) |
| --- | --- |
| Which of the following best describes you? |  |
| Female | 97 (63%) |
| Male | 57 (37%) |
| What is your ethnic group? |  |
| White | 175 (51%) |
| Black | 0 (0%) |
| Asian | 0 (0%) |
| Mixed or multiple ethnic groups | 3 (2%) |
| Other | 1 (0%) |
| Missing | 75 (47%) |
| Please indicate how you relate to the kidney transplantation clinical journey. |  |
| Donor | 12 (4%) |
| Patient | 48 (16%) |
| Transplant surgeon | 35 (13%) |
| Nephrologist | 93 (33%) |
| Transplant coordinator | 20 (7%) |
| Hospital administrative | 19 (7%) |

**Section 2 – Chronic Kidney Disease**

| 1. In your opinion, how consistent or similar is the definition and classification of chronic kidney disease across health care providers? | N | % |
| --- | --- | --- |
| Not consistent at all | 0 | 0 |
| Slightly consistent | 18 | 9 |
| Moderately consistent | 74 | 38 |
| Very consistent | 86 | 44 |
| Extremely consistent | 14 | 7 |
| Total | **193** |  |
| 1. Please identify which of the following risk factors would likely result in testing a patient for chronic kidney disease by using estimated glomerular filtration rate (eGFR), creatinine and albumin-to-creatinine ratio. | **N** | **%** |
| Diabetes | 128 | 70 |
| Hypertension | 127 | 69 |
| Family history of end-stage kidney disease or hereditary kidney disease | 112 | 61 |
| Age | 107 | 58 |
| Acute kidney injury | 98 | 54 |
| Multisystem diseases with potential kidney involvement | 107 | 58 |
| Obstructive uropathy | 97 | 53 |
| Recurrent kidney stones | 94 | 51 |
| Cardiovascular disease | 96 | 52 |
| Prostatic hypertrophy | 43 | 23 |
| Race/ethnicity | 35 | 19 |
| Gender | 30 | 16 |
| Total | **183** |  |
| 1. Please identify whether you had any of the following conditions prior to developing chronic kidney disease | **N** | **%** |
| Hypertension | 12 | 22 |
| Family history of end-stage kidney disease (glomerular filtration rate (gfr) category g5) or hereditary kidney disease | 8 | 15 |
| Acute kidney injury | 7 | 13 |
| Diabetes | 5 | 9 |
| Recurrent kidney stones | 5 | 9 |
| Multisystem diseases with potential kidney involvement | 5 | 9 |
| Prostatic hypertrophy | 5 | 9 |
| Cardiovascular disease | 5 | 9 |
| Obstructive uropathy | 3 | 5 |
| Total | **55** |  |
| 1. Once a patient is diagnosed with chronic kidney disease, how easy is it to access resources such as patient groups and help with health information? | **N** | **%** |
| Extremely difficult | 6 | 4 |
| Somewhat difficult | 33 | 20 |
| Neither easy nor difficult | 42 | 25 |
| Somewhat easy | 71 | 42 |
| Extremely easy | 6 | 4 |
| Total | **169** |  |
| 1. Please indicate which health care professionals are involved in the regular care of patients newly diagnosed with chronic kidney disease. | **N** | **%** |
| GP | 103 | 56 |
| Specialist nurse | 84 | 45 |
| Medical specialist | 175 | 95 |
| Transplant coordinator | 29 | 16 |
| Total | **185** |  |
| 1. Please indicate how patient education on nutrition and lifestyle changes is available for newly diagnosed patients. | **N** | **%** |
| A dedicated staff member in the centre | 103 | 58 |
| Referral to patient educators in centre | 63 | 35 |
| Instructions on how to find materials online for patient education | 57 | 32 |
| Referral to kidney disease societies in centre | 25 | 14 |
| Ehealth or mhealth solutions | 5 | 3 |
| Total | **179** |  |
| 1. In your opinion, how appropriate is the frequency of monitoring according to estimated glomerular filtration rate (eGFR) and albuminuria categories? | **N** | **%** |
| Adequate | 32 | 19 |
| Somewhat infrequent | 36 | 22 |
| Somewhat too frequent | 18 | 11 |
| Far too infrequent | 18 | 11 |
| Total | **165** |  |
| 1. In your opinion, how appropriate is the frequency of monitoring according to estimated glomerular filtration rate (eGFR) and albuminuria categories? | **N** | **%** |
| Glomerular filtration rate (gfr)< 20 | 47 | 31 |
| Glomerular filtration rate (gfr)< 30 | 52 | 35 |
| Progressive and irreversible chronic kidney disease over the preceding 6-12 months | 46 | 31 |
| Chronic kidney disease diagnosis | 55 | 37 |
| End stage kidney disease diagnosis | 38 | 25 |
| National guidelines that use different criteria (please specify the criteria) | 5 | 3 |
| Total | **150** |  |
| 1. When is a patient told about kidney transplant options, after an end stage kidney disease diagnosis? | **N** | **%** |
| Immediately | 78 | 53 |
| Within 1 to 6 months | 37 | 25 |
| Between 6 months and 1 year | 8 | 5 |
| More than 1 year after | 9 | 6 |
| Total | **146** |  |
| 1. When first addressing the possibility of a kidney transplant, how much do you rely on potential living donors and on deceased donors? | **N** | **%** |
| Both living and deceased donation | 117 | 78 |
| Mostly living donation | 11 | 7 |
| Mostly deceased donation | 16 | 11 |
| Only deceased donation | 8 | 5 |
| Total | **150** |  |
| 1. In your institution, how easily accessible is the information that is aimed to be given to a patient in preparation for a kidney transplant? | **N** | **%** |
| Extremely accessible | 24 | 16 |
| Very accessible | 54 | 36 |
| Moderately accessible | 47 | 31 |
| Slightly accessible | 5 | 3 |
| Not accessible at all | 1 | 1 |
| Total | **151** |  |
| 1. If transplant-related education is available to patients, who is responsible to provide it? | **N** | **%** |
| A designated healthcare professional in the centre educates our patients | 77 | 53 |
| I personally participate in the education each of our patients | 79 | 54 |
| We refer patients to patient educators in our centre | 31 | 21 |
| We tell patients how to find materials online for their education | 25 | 17 |
| We refer patients to kidney disease societies in our centre for their education | 30 | 21 |
| We use ehealth or mhealth solutions | 7 | 48 |
| Total | **146** |  |
| 1. If education before a kidney transplant is provided, which topics are covered in the education? | **N** | **%** |
| Importance of the adherence to medication | 138 | 93 |
| The reasons behind all of the tests needed in preparation for the transplant | 128 | 86 |
| Potential complications from a surgery and/or medication side effects | 121 | 82 |
| How the test results influence clinical decisions | 103 | 70 |
| Timelines to recovery | 89 | 60 |
| How to emotionally prepare for surgery | 52 | 35 |
| Creating a support structure for post-surgical recovery | 43 | 29 |
| The impacts of cytomegalovirus (CMV) and other infections to transplant | 58 | 39 |
| Total | **147** |  |
| 1. Please indicate which of the following are available at your centre for patients who choose not to pursue any form of renal replacement therapy. | **N** | **%** |
| Psychological care | 61 | 47 |
| Protocols for symptom and pain management | 59 | 45 |
| Coordinated end-of-life care | 81 | 62 |
| Culturally sensitive care for the patient and their family (home | 32 | 24 |
| Spiritual care | 30 | 23 |
| Total | **131** |  |
| 1. How effective is the support from patients' associations in the run up to a kidney transplant? | 4.64/10 | |
|  |  | |
| 1. Is Cytomegalovirus (CMV) testing a pre-transplant requirement at your centre? | **N** | **%** |
| CMV serology for transplant recipients | 74 | 54 |
| CMV serology for donors | 72 | 53 |
| CMV molecular testing for donors | 24 | 18 |
| CMV molecular testing for transplant recipients | 22 | 16 |
| Total | **137** |  |
| 1. What are the 4 most common reasons a patient may be deemed unsuitable for transplantation at your centre? | **N** | **%** |
| Cancer | 75 | 74 |
| Severity of co-morbidities | 69 | 68 |
| Peripheral vascular disease | 44 | 44 |
| Overall health status | 37 | 36 |
| Body mass index | 41 | 41 |
| Substance abuse | 36 | 36 |
| Autoimmune disease severity | 16 | 16 |
| Mental health challenges | 19 | 19 |
| Advanced age | 19 | 19 |
| Multiple previous transplants | 7 | 7 |
| Current medication schedule conflicts with post-transplant additions | 4 | 4 |
| Highly sensitised patient | 4 | 4 |
| Smoking | 2 | 2 |
| Previous failed transplant | 1 | 1 |
| Total | **101** |  |
| 1. In your centre, how consistent or similar are kidney transplant suitability criteria? | **N** | **%** |
| Extremely consistent | 16 | 11 |
| Very consistent | 62 | 43 |
| Moderately consistent | 43 | 30 |
| Slightly consistent | 2 | 1 |
| Not consistent at all | 1 | 1 |
| Total | **145** |  |
| 1. In your centre, which specialties regularly interact to coordinate care for a patient in preparation for surgery? | **N** | **%** |
| Nephrologist | 131 | 89 |
| Transplant surgeons | 102 | 78 |
| Specialised nurse | 76 | 58 |
| Urologist | 88 | 67 |
| Immunologist | 82 | 63 |
| Psychologist/psychiatrist | 61 | 47 |
| Cardiologist | 64 | 49 |
| Pathologist | 35 | 27 |
| Primary care physician | 27 | 21 |
| Patient educators | 15 | 11 |
| Social worker | 15 | 11 |
| Patient advocates | 3 | 2 |
| Total | **147** |  |
| 1. In your opinion, how effective is the coordination and communication among all of the specialties to monitor a patient with chronic kidney disease at your centre? | **N** | **%** |
| Extremely effective | 12 | 8 |
| Very effective | 73 | 48 |
| Moderately effective | 43 | 28 |
| Slightly effective | 13 | 9 |
| Not effective at all | 2 | 1 |
| Total | **151** |  |
| 1. In your opinion, how effective is the coordination and communication among all of the specialties to prepare a patient for a kidney transplant at your centre? | **N** | **%** |
| Extremely effective | 14 | 9 |
| Very effective | 74 | 48 |
| Moderately effective | 53 | 35 |
| Slightly effective | 11 | 7 |
| Not effective at all | 1 | 1 |
| Total | **153** |  |

**Section 3 - Kidney donation and transplantation**

| Kidney donation and Tx Qs |  | |
| --- | --- | --- |
| 22) In your opinion, how effective are referral systems of potential deceased donors, in your centre? | **N** | **%** |
| Extremely effective | 17 | 13 |
| Very effective | 58 | 45 |
| Moderately effective | 52 | 40 |
| Slightly effective | 5 | 4 |
| Not effective at all | 1 | 1 |
| Total | **129** |  |
| 23) In your opinion, how effective are referral systems of potential donation after circulatory death (DCD) in your centre? | **N** | **%** |
| Extremely effective | 18 | 14 |
| Very effective | 45 | 35 |
| Moderately effective | 37 | 29 |
| Slightly effective | 12 | 9 |
| Not effective at all | 8 | 6 |
| Total | **129** |  |
| 24) How much does the referral of donation after brain death (DBD) rely on well-defined clinical criteria? | **N** | **%** |
| Extremely reliant | 22 | 17 |
| Very reliant | 71 | 56 |
| Moderately reliant | 31 | 24 |
| Slightly reliant | 2 | 2 |
| Not reliant at all | 1 | 1 |
| Total | **127** |  |
| 25) How much does the referral of donation after circulatory death (DCD) rely on well-defined clinical criteria? | **N** | **%** |
| Extremely reliant | 21 | 18 |
| Very reliant | 61 | 53 |
| Moderately reliant | 30 | 26 |
| Slightly reliant | 3 | 3 |
| Not reliant at all | 1 | 1 |
| Total | **116** |  |
| 26) In your opinion, how effective is in your centre the coordination with intensive care unit to enable organ donation? | **N** | **%** |
| Extremely effective | 25 | 19 |
| Very effective | 62 | 46 |
| Moderately effective | 34 | 25 |
| Slightly effective | 7 | 5 |
| Not effective at all | 4 | 3 |
| Total | **135** |  |
| 27) How often is organ donation incorporated in intensive care end of life care in patients with severe brain injury? | **N** | **%** |
| Most of the time | 74 | 57 |
| About half the time | 25 | 19 |
| Sometimes | 25 | 19 |
| Always | 32 | 25 |
| Never | 3 | 2 |
| 28) Consider the determination of death of potential donors, either brain (DBD) or circulatory (DCD). Please identify which of the following limitations apply to your regular practice. | **N** | **%** |
| Failure to identify/refer a potential donor | 34 | 29 |
| Reluctance of families to accept circulatory death | 35 | 30 |
| Does not apply to me | 33 | 28 |
| Circulatory death not declared within the appropriate timeframe | 27 | 23 |
| Brain death diagnosis not confirmed (i.e. does not fulfil criteria) | 18 | 15 |
| Lack of appropriate space to withdraw treatment or other logistical problems | 12 | 10 |
| Reluctance of healthcare professionals to accept circulatory death | 7 | 6 |
| Brain death diagnosis not completed due to lack of clinician to make diagnosis | 2 | 1 |
| Brain death diagnosis not completed due to lack of technical resources to perform confirmatory tests | 2 | 1 |
| Brain death diagnosis not completed due to lack of clinician to perform confirmatory tests | 2 | 1 |
| 29) With regards to protocols for determination of death of potential donation after brain death (DBD), please identify which of the following apply to your regular practice. | **N** | **%** |
| National protocols | 88 | 74 |
| Regional protocols | 26 | 22 |
| Local protocols | 28 | 24 |
| Total | **119** |  |
| 30) With regards to protocols for determination of potential donation after brain death (DBD), please identify which of the following are involved in defining these protocols. | **N** | **%** |
| Donation/transplantation authority | 73 | 63 |
| Donor coordinators | 65 | 56 |
| Local clinicians | 49 | 43 |
| Organ procurement teams | 41 | 36 |
| Total | **115** |  |
| 31) In your centre, how long is the standard no touch period after cardiac arrest, in the case of donation after circulatory death (DCD)? | **N** | **%** |
| >20 minutes | 29 | 48 |
| 10-20 minutes | 8 | 13 |
| 5-10 minutes | 13 | 22 |
| 2-5 minutes | 9 | 15 |
| Total | **59** |  |
| 32) In your centre, where does withdrawal of therapy take place in the case of donation after circulatory death (DCD)? | **N** | **%** |
| Intensive care unit | 58 | 92 |
| In theatre | 24 | 38 |
| In anesthetic room | 4 | 6 |
| Total | **63** |  |
| 33) In your opinion, how timely is a potential deceased donor's family first contact, at your centre? | **N** | **%** |
| Extremely early | 12 | 10 |
| Somewhat early | 15 | 13 |
| Adequate | 80 | 69 |
| Somewhat late | 7 | 6 |
| Total | **116** |  |
| 34) In your opinion, how adequate is the communication training for approaching a potential deceased donor's family, at your centre? | **N** | **%** |
| Extremely adequate | 8 | 7 |
| Very adequate | 61 | 51 |
| Moderately adequate | 44 | 37 |
| Slightly adequate | 3 | 3 |
| Not adequate at all | 1 | 1 |
| Total | **119** |  |
| 35) In your opinion, how effective is the coordination and communication between donor coordinators and other health professionals at your centre? | **N** | **%** |
| Extremely effective | 10 | 11 |
| Very effective | 46 | 51 |
| Moderately effective | 30 | 33 |
| Slightly effective | 3 | 3 |
| Not effective at all | 1 | 1 |
| Total | **90** |  |
| 36) In your opinion, how adequate are the protocols for obtaining family consent for donation after circulatory death (DCD) at your centre? | **N** | **%** |
| Extremely adequate | 7 | 10 |
| Very adequate | 30 | 41 |
| Moderately adequate | 25 | 34 |
| Slightly adequate | 6 | 8 |
| Not adequate at all | 5 | 7 |
| Total | **74** |  |
| 37) Consider all the professionals involved in the management of potential donors after brain death (DBD), at your centre. Is the assignment of roles clear? | 7.0/10 | |
|  |  | |
| 38) In your opinion, how adequate are the protocols for monitoring and optimising potential donors after brain death (DBD) at your centre? | **N** | **%** |
| Extremely adequate | 10 | 9 |
| Very adequate | 67 | 58 |
| Moderately adequate | 33 | 29 |
| Slightly adequate | 3 | 2 |
| Not adequate at all | 1 | 1 |
| Total | **115** |  |
| 39) In your opinion, how adequate are the protocols to optimise the timing to perform organ procurement from a deceased donor at your centre? | **N** | **%** |
| Extremely adequate | 9 | 8 |
| Very adequate | 57 | 50 |
| Moderately adequate | 35 | 31 |
| Slightly adequate | 12 | 11 |
| Not adequate at all | 1 | 1 |
| Total | **114** |  |
| 40) How would you rate the willingness to take risks in living donor - recipient match, in your regular practice? | 7.14/10 | |
|  |  | |
| 41) How would you rate the willingness to take risks in deceased donor - recipient match, in your regular practice? | 6.14/10 | |
|  |  | |
| 42) How would you rate the willingness to consider high risk living donors, in your regular practice? | 4.51/10 | |
|  |  | |
| 43) How would you rate the willingness to consider high risk deceased donors, in your regular practice? | 6.18/10 | |
|  |  | |
| 44) Specifically, please identify which of the following donor related criteria could lead you to potentially decline a deceased donor offer. | **N** | **%** |
| Neoplastic disease | 99 | 88 |
| High risk donor | 46 | 41 |
| HIV positive | 47 | 42 |
| Age threshold | 24 | 21 |
| Risk taking behaviours | 23 | 20 |
| HbsAg HBV positive | 38 | 34 |
| BMI threshold | 11 | 10 |
| Hepatitis c virus (HCV) positive | 20 | 18 |
| Extended criteria donor | 14 | 12 |
| Cytomegalovirus (CMV) positive | 7 | 6 |
| HBV Ab-core positive | 11 | 10 |
| Total | **112** |  |
| 45) Do you trigger prophylactic hepatitis treatment when accepting a deceased donor at high risk of infectious decease offer? | **N** | **%** |
| Yes | 66 | 59 |
| No | 38 | 41 |
| Total | **94** |  |
| 46) Does a national organ allocation policy influence your donor selection criteria? | 4.82/10 | |
|  |  | |
| 47) How frequently are deceased donor allocation algorithms used, in your centre? | **N** | **%** |
| Always | 64 | 61 |
| Most of the time (no specific patient criteria) | 22 | 21 |
| About half the time (no specific patient criteria) | 7 | 7 |
| Sometimes (no specific patient criteria) | 6 | 6 |
| Only for highly sensitised patients | 9 | 9 |
| Never | 3 | 3 |
| Total | **105** |  |
| 48) Please identify which of the following kidney donor criteria could lead you to potentially decline a deceased donor offer. | **N** | **%** |
| Biopsy findings | 82 | 75 |
| Donor-recipient age mismatch | 50 | 46 |
| Cold ischemia time | 52 | 48 |
| Diabetes mellitus (type 1 or 2) | 30 | 21 |
| Abnormal albumin-to-creatinine ratio | 44 | 40 |
| Absence of imaging | 18 | 17 |
| Donor age | 22 | 20 |
| History of acute kidney injury | 20 | 18 |
| Use of street drugs | 22 | 21 |
| Limited history of renal function | 45 | 41 |
| Other risky personal behaviour | 15 | 14 |
| Total | **109** |  |
| 49) How do you weigh in risks of disease (infection, cancer) transmission with risk from the transplant candidate not receiving an organ offer on time? | **N** | **%** |
| Follow the guidelines | 87 | 84 |
| Based on discussion with transplant candidate | 37 | 36 |
| Based on clinical experience | 32 | 31 |
| It's the transplant candidate's decision | 9 | 9 |
| Total | **104** |  |
| 50) In your opinion, how accessible is a transplant coordinator at your centre? | **N** | **%** |
| Extremely accessible | 31 | 25 |
| Very accessible | 56 | 46 |
| Moderately accessible | 28 | 28 |
| Slightly accessible | 6 | 5 |
| Not accessible at all | 1 | 1 |
| Total | **122** |  |
| 51) How readily available are procurement teams at your centre? | 8.29/10 | |
|  | | |
|  |  | |
| 52) In your opinion, how effective is the coordination and communication between organ-specific retrieval teams, in your centre? | **N** | **%** |
| Extremely effective | 20 | 20 |
| Very effective | 60 | 53 |
| Moderately effective | 26 | 23 |
| Slightly effective | 5 | 5 |
| Total | **111** |  |
| 53) How would you rate average cold ischemia time of deceased donor offers at your centre? | 6.02/10 | |
|  |  | |
| 54) Please identify which techniques for packaging and transportation are regularly used at your centre. | **N** | **%** |
| Static cold storage | 67 | 61 |
| Hypothermic machine perfusion starting on donor hospital site | 33 | 30 |
| Hypothermic machine perfusion starting on recipient hospital site | 26 | 24 |
| Assessment and reconditioning at transplant site | 27 | 35 |
| Normothermic machine perfusion on recipient hospital site | 13 | 12 |
| Normothermic machine perfusion starting on donor hospital site | 5 | 5 |
| Total | **109** |  |
| 55) In your opinion, how effective are consent safeguards in living donation in your centre? | **N** | **%** |
| Extremely effective | 32 | 31 |
| Very effective | 60 | 59 |
| Moderately effective | 21 | 21 |
| Slightly effective | 3 | 3 |
| Not effective at all | 1 | 1 |
| Total | **102** |  |
| 56) In your opinion, how effective are kidney paired exchanges your centre is involved in? | **N** | **%** |
| Extremely effective | 15 | 13 |
| Moderately effective | 43 | 37 |
| Very effective | 37 | 32 |
| Slightly effective | 11 | 10 |
| Not effective at all | 6 | 6 |
| Total | **115** |  |
| 57) In your opinion, how effective is the unspecified (or non-directed) living donor registry at your centre? | **N** | **%** |
| Extremely effective | 10 | 10 |
| Moderately effective | 30 | 29 |
| Very effective | 32 | 31 |
| Slightly effective | 12 | 12 |
| Not effective at all | 4 | 4 |
| Total | **104** |  |
| 58) Please identify which of the following donor criteria could lead you to potentially decline a living donor | **N** | **%** |
| Neoplastic disease | 95 | 86 |
| High risk donor | 74 | 67 |
| HIV positive | 53 | 48 |
| BMI threshold | 58 | 52 |
| Use of street drugs | 57 | 51 |
| Age threshold | 34 | 31 |
| Other risk-taking behaviours | 31 | 28 |
| Hepatitis C virus (HCV) positive | 37 | 33 |
| Extended criteria donor | 25 | 22 |
| HBV positive | 31 | 28 |
| Cytomegalovirus (CMV) positive | 2 | 2 |
| Total | **111** |  |
| 59) How would you rate the kidney donation and transplantation biovigilance and surveillance system in your centre? | 7.18/10 | |
|  |  | |
| 60) In your opinion, how adequate is the collection of performance and quality indicators on kidney donation, either living or deceased, in your centre? | **N** | **%** |
| Extremely adequate | 36 | 32 |
| Somewhat adequate | 53 | 48 |
| Neither adequate nor inadequate | 8 | 7 |
| Somewhat inadequate | 9 | 8 |
| Extremely inadequate | 5 | 5 |
| Total | **112** |  |
| 61) In your opinion, how adequate is the collection of performance and quality indicators on kidney transplantation, in your centre? | **N** | **%** |
| Extremely adequate | 35 | 43 |
| Somewhat adequate | 39 | 48 |
| Neither adequate nor inadequate | 5 | 6 |
| Somewhat inadequate | 2 | 2 |
| Total | **81** |  |
| 62) Consider your centre's donation and transplantation end-point data. How easily accessible is it? | 6.63/10 | |
|  |  | |
| 63) In your opinion, how effective are the protocols to monitor risk adjustment variables, in your centre? | **N** | **%** |
| Extremely effective | 11 | 11 |
| Very effective | 47 | 46 |
| Moderately effective | 38 | 37 |
| Slightly effective | 7 | 7 |
| Total | **103** |  |

**Section 4 – Transplant Recipients**

| Tx recipients Qs |  | |
| --- | --- | --- |
| 64) In your opinion, how adequate is the time that it takes for a transplant recipient to start post-op rehabilitation, in your centre? | **N** | **%** |
| Extremely adequate | 31 | 29 |
| Somewhat adequate | 54 | 51 |
| Somewhat inadequate | 6 | 6 |
| Neither adequate nor inadequate | 9 | 8 |
| Total | **106** |  |
| 65) Please identify which healthcare professionals are involved in a transplant recipient's short term (in hospital and up to one-month post-transplant) follow up. | **N** | **%** |
| Nephrologist | 106 | 95 |
| Transplant surgeon | 70 | 63 |
| Urologist | 68 | 61 |
| Infectious disease specialist | 45 | 41 |
| Immunologist | 44 | 40 |
| Transplant coordinator | 23 | 21 |
| Endocrinologist | 24 | 22 |
| Primary care physician | 25 | 22 |
| Cardiologist | 18 | 16 |
| Pathologist | 23 | 21 |
| Total | **111** |  |
| 66) Please identify which health professionals are involved in a transplant recipient's long term (after the first month) follow up. | **N** | **%** |
| Nephrologist | 112 | 99 |
| Transplant surgeon | 36 | 32 |
| Primary care physician | 44 | 39 |
| Transplant coordinator | 18 | 16 |
| Immunologist | 26 | 26 |
| Cardiologist | 21 | 19 |
| Infectious disease specialist | 19 | 17 |
| Urologist | 29 | 26 |
| Pathologist | 13 | 12 |
| Endocrinologist | 13 | 12 |
| Total | **113** |  |
| 67) In your opinion, how effective is the coordination and communication among all of the specialties in referring transplant patients back to primary care, at your centre? | **N** | **%** |
| Extremely effective | 10 | 9 |
| Very effective | 50 | 47 |
| Moderately effective | 29 | 27 |
| Slightly effective | 13 | 12 |
| Not effective at all | 4 | 4 |
| Total | **106** |  |
| 68) How effective is the coordination and communication among all of the specialties with regards to the patient's transplant medications? | **N** | **%** |
| Extremely effective | 17 | 15 |
| Very effective | 51 | 46 |
| Moderately effective | 32 | 29 |
| Slightly effective | 7 | 6 |
| Not effective at all | 2 | 2 |
| Total | **112** |  |
| 69) How effective is the support from patients' associations after a kidney transplant? | **N** | **%** |
| Extremely effective | 4 | 4 |
| Very effective | 37 | 36 |
| Moderately effective | 35 | 34 |
| Slightly effective | 21 | 21 |
| Not effective at all | 4 | 4 |
| Total | **102** |  |
| 70) Please identify which Cytomegalovirus (CMV) prevention protocols are most in use at your centre. | **N** | **%** |
| Mostly pre-emptive | 25 | 23 |
| Based on the patient's risk profile | 47 | 43 |
| Equally prophylactic and pre-emptive | 36 | 33 |
| Mostly prophylactic | 20 | 16 |
| Total | **109** |  |
| 71) When a kidney transplant results in graft failure at your centre, what are the three most common causes? | **N** | **%** |
| Chronic rejection | 64 | 79 |
| Recurrent disease | 32 | 40 |
| Acute rejection | 40 | 49 |
| Non-adherence | 32 | 40 |
| Infection | 22 | 27 |
| Donor kidney problems | 23 | 28 |
| Clotting (any) | 12 | 15 |
| Medication side effects | 13 | 16 |
| Fluid collection | 3 | 4 |
| Total | **81** |  |
| 72) Please characterise the frequency of post-transplant follow-up visits, according to your protocol or standard practice for the first 12 months. | **N** | |
| First month | | |
| Three times per week | 41 | |
| Two times per week | 36 | |
| Once per week | 35 | |
| Once or twice per month | 17 | |
| Lower frequency | 1 | |
| Not applicable | 3 | |
| 2-3 months | | |
| Three times per week | 1 | |
| Two times per week | 18 | |
| Once per week | 38 | |
| Once or twice per month | 42 | |
| Lower frequency | 1 | |
| Not applicable | 1 | |
| More than 3 months post-transplant | | |
| Three times per week | 0 | |
| Two times per week | 1 | |
| Once per week | 12 | |
| Once or twice per month | 36 | |
| Lower frequency | 65 | |
| Not applicable | 7 | |
| 73) Is the frequency of blood tests the same as follow up visits? | **N** | **%** |
| Yes | 5 | 71 |
| No, lower | 1 | 14 |
| No, higher | 1 | 14 |
| Total | **7** |  |
| 74) Are patient quality of life (QOL) outcomes collected at your centre? | **N** | **%** |
| No, never | 49 | 43 |
| Sometimes | 50 | 44 |
| Yes, always | 15 | 13 |
| Total | **114** |  |
| 75) Are Patient Reported Outcome Measure (PROMS) collected at your centre? | **N** | **%** |
| No, never | 54 | 49 |
| Sometimes | 43 | 39 |
| Yes, always | 14 | 13 |
| Total | **111** |  |
| 76) Are Donor Reported Outcome Measure (DROMS) routinely collected at your centre? | **N** | **%** |
| No, never | 60 | 55 |
| Sometimes | 33 | 30 |
| Yes, always | 16 | 15 |
| Total | **109** |  |
| 77) How would you rate your overall donor experience of living kidney donation? | 6.84/10 | |
|  |  | |
| 78) Based on your experience with living kidney donation, would you recommend it to others? | 7.85/10 | |
|  |  | |
| 79) How would you rate your likelihood to donate your organs after death? | 8.75/10 | |
|  |  | |
| 80) How would you rate wound-related pain that you are experiencing (if applicable) when you are just resting? | 7.63/10 | |

**Section 5 – Management of Donation and Transplant Services**

| Management of donation and Tx service |  | |
| --- | --- | --- |
| 81) To what degree do reimbursement policies and practices influence kidney transplantation clinical decision making in your centre? | **N** | **%** |
| Significantly: There are tests and procedures we are unable to conduct that are necessary in other countries. | 1 | 1 |
| Partially: we are able to do most everything we would like to. | 34 | 39 |
| Not at all: clinical decisions are not impacted in the preparation for kidney transplant. | 52 | 60 |
| Total | **87** |  |
| 82) In your opinion, how much do reimbursement rates drive how resources are utilised, in your centre? | 3.95/10 | |
|  |  | |
| 83) Consider the financial support available for patients, in your centre. In your opinion, how adequate is it? |  | |
| Patients undergoing dialysis | 5.93/10 | |
| Transplant recipients, pre-transplant | 5.72/10 | |
| Transplant recipients, post-transplant | 6.06/10 | |
| 84) In your opinion, how adequate is the reimbursement of the Diagnosis related group (DRG) system in your centre to cover the extent of expenses incurred at your centre for kidney transplant and post-transplant care including the needed pharmaceuticals for reimbursement? | **N** | **%** |
| Extremely adequate | 14 | 22 |
| Somewhat adequate | 23 | 35 |
| Somewhat inadequate | 16 | 25 |
| Neither adequate nor inadequate | 9 | 14 |
| Extremely inadequate | 3 | 5 |
| Total | **65** |  |
| 85) Do primary care or specialty providers receive any financial incentives and/or penalties for pre-set targets? and/or penalties occur? | **N** | **%** |
| Financial incentives and/or penalties for operational targets (e.g. wait times) | 18 | 37 |
| Financial incentives and/or penalties for economic and cost savings targets (e.g. generic prescribing) | 9 | 18 |
| Financial incentives and/or penalties for patient-generated or patient reported outcomes targets (e.g. patient satisfaction) | 17 | 35 |
| Financial incentives and/or penalties for clinical outcomes targets (e.g. mortality rates) | 5 | 10 |
| Total | **49** |  |
| 86) How are electronic medical records currently being used in kidney donation and transplantation? | **N** | **%** |
| Tracking of patient health history | 81 | 41 |
| Improvement of quality through the use of clinical decision-support | 44 | 22 |
| Not currently used (patient data is recorded on paper) | 21 | 11 |
| Analytical capabilities for population health such as patient risk stratification and/or predictive modeling | 33 | 11 |
| Total | **199** | 17 |
| 87) In your opinion, how effective is the health authority for transplantation in regulating innovation, procurement, and financing? | **N** | **%** |
| Extremely effective | 6 | 6 |
| Very effective | 26 | 25 |
| Moderately effective | 53 | 51 |
| Slightly effective | 18 | 17 |
| Total | **103** |  |
